# Ferroptosis-related signature and immune infiltration characterization in acute lung injury/acute respiratory distress syndrome

**DOI:** 10.1186/s12931-023-02429-y

**Published:** 2023-06-10

**Authors:** Aijia Ma, Zhongxue Feng, Yang Li, Qin Wu, Huaiyu Xiong, Meiling Dong, Jiangli Cheng, Zhenling Wang, Jing Yang, Yan Kang

**Affiliations:** 1 Department of Critical Care Medicine, West China Hospital of Sichuan University, No. 37, Guoxue Alley, Chengdu, 610041 Sichuan China; 2 Department of Critical Care Medicine, State Key Laboratory of Biotherapy and Cancer Center, West China Hospital, Sichuan University and Collaborative Innovation Center of Biotherapy, Chengdu, Sichuan China

## Abstract

**Background:**

Acute lung injury/acute respiratory distress syndrome (ALI/ARDS) is one of the most life-threatening diseases in the intensive care unit with high mortality and morbidity. Ferroptosis is a newly discovered immune related cell death that is associated with various lung diseases. However, the role of immune-mediated ferroptosis in ALI/ARDS has not been elucidated.

**Method:**

We analyzed two Gene Expression Omnibus (GEO) datasets (GSE2411 and GSE109913) and extracted characteristic ferroptosis-related genes (FRGs) between the control and ALI groups through bioinformatic analysis. Then, we prospectively collected bronchoalveolar lavage fluid (BALF) from patients with ARDS and verified the expression of characteristic FRGs. Lastly, we constructed the ALI/ARDS model induced by LPS and isolated the primary neutrophils of mice. Erastin, an ferroptosis inducer, was used at the cellular level to verify the effect of neutrophils on ferroptosis in lung epithelium cells.

**Result:**

We identified three characteristic FRGs, Cp, Slc39a14 and Slc7a11, by analyzing two gene expression profiling datasets. Immune infiltration analysis showed that the three characteristic genes were significantly positively correlated with the infiltration levels of neutrophils. We collected BALF from 59 ARDS patients to verify the expression of Cp, Slc7a11 and Slc39a14 in humans. The results showed that Cp was elevated in patients with severe ARDS (*p* = 0.019), Slc7a11 was significantly elevated in patients with moderate ARDS (*p* = 0.021) relative to patients with mild ARDS. The levels of neutrophils in the peripheral blood of ARDS patients were positively correlated with the expression levels of Slc7a11 (Pearson’s R^2^ = 0.086, *p* = 0.033). Three characteristic FRGs were significantly activated after the onset of ferroptosis (6 h) early in LPS induced ALI model, and that ferroptosis was alleviated after the organism compensated within 12 to 48 h. We extracted primary activated neutrophils from mice and co-cultured them with MLE-12 in transwell, Slc7a11, Cp and Slc39a14 in MLE-12 cells were significantly upregulated as the number of neutrophils increased. The results showed that neutrophil infiltration alleviated erastin-induced MDA accumulation, GSH depletion, and divalent iron accumulation, accompanied by upregulation of Slc7a11 and Gpx4, implying the existence of a compensatory effect of lipid oxidation in neutrophils after acute lung injury in the organism.

**Conclusion:**

We identified three immune-mediated ferroptosis genes, namely, Cp, Slc7a11 and Slc39a14, which possibly regulated by neutrophils during the development of ALI, and their pathways may be involved in anti-oxidative stress and anti-lipid metabolism. Thus, the present study contributes to the understanding of ALI/ARDS and provide novel targets for future immunotherapeutic.

**Supplementary Information:**

The online version contains supplementary material available at 10.1186/s12931-023-02429-y.

## Introduction

Acute lung injury/acute respiratory distress syndrome (ALI/ARDS) is a serious lung disease characterized by refractory hypoxemia and noncardiogenic pulmonary edema [1, 2]. Its morbidity and mortality are increasing with more than 3 million cases and 75,000 deaths worldwide each year [3]. In the last few years, despite extensive research trying to identify various effective targeted drugs to treat ALI/ARDS, there is still a 40–60% mortality rate in the intensive care unit [4].

The high mortality of ARDS lies not only in the difficulty of correcting oxygenation but also in the difficulty of stabilizing its dynamically changing immune status. Massive diffuse damage to lung epithelial and endothelial cells activates a strong systemic inflammatory and immune response in ALI/ARDS. A variety of immune cells processes, such as neutrophil infiltration, differentiation of Treg cells, and M1/M2 polarization of macrophages, play an important role in regulating immune homeostasis during the development of ALI/ARDS [5–7]. Therefore, immunomodulatory mechanisms in ALI/ARDS warrant further investigation and may be a potential direction for future interventions in ALI/ARDS.

Ferroptosis is an iron-dependent form of regulated cell death [8]. The ferroptosis process involves inhibition of the antioxidant system, including a reduction in glutathione peroxidase 4 (Gpx4) activity and a depletion of glutathione (GSH), which leads to an accumulation of deadly levels of lipid peroxides that contribute to cellular pathogenesis. GSH/Gpx4 axis plays an important antioxidant role in the regulation of lipid peroxidation produced by ferroptosis [8]. The significance of ferroptosis and iron metabolism in ALI/ARDS has been highlighted in recent years [9]. The levels of iron and iron-related proteins found in the bronchoalveolar lavage fluid (BALF) correlate with the severity of ARDS [10]. More importantly, ferroptosis is considered an immune-derived cell death and is immune-regulated [11, 12]. Additionally, ferroptosis is linked to the sustained release of inflammatory cytokines and damage-associated molecular patterns (DAMPs), which stimulate a series of systemic inflammatory processes and amplify organ damage [13]. Pulmonary edema and alveolar inflammation with high levels of cytokines (IL-1β, IL-6 and TNF-α) are aggravated by ferroptosis, which can be alleviated by ferroptosis inhibitors [14].

However, although there is sufficient evidence for an association of ALI/ARDS with immune infiltration and ferroptosis, the relationship among all three is unknown. Thus, understanding the interactions between lung immune cell infiltration and ferroptosis may provide new strategies for ARDS treatment, such as ferroptosis-based immunotherapy.

This study identified characteristic ferroptosis-related genes (FRGs) associated with immune infiltration in ALI/ARDS by analyzing two GEO datasets. The expression levels of these genes were confirmed in human bronchoalveolar lavage fluid (BALF) from ARDS patients of varying severity, and correlated with neutrophil levels. Animal and cellular models revealed a compensatory mechanism for lung epithelial ferroptosis through neutrophil infiltration, providing new insights into the interplay between ferroptosis and the immune response in ALI/ARDS, thereby introducing a potential new target for the subsequent treatment of ALI/ARDS.

## Methods

### Data acquisition and ferroptosis-related genes

The GSE2411 [15] and GSE109913 microarray datasets were downloaded from the Gene Expression Omnibus database (GEO) of the NCBI database (https://www.ncbi.nlm.nih.gov/). The GSE2411 dataset was generated using the Illumina HiSeq 4000 (Mus musculus) platform, and the GSE109913 dataset was generated using the Affymetrix Mouse Expression 430A Array platform. Because GEO databases are publicly available, ethical approval was not required for the present study. Corresponding ferroptosis-related genes were downloaded from the KEGG database (https://www.kegg.jp/keggbin/show_pathway/mmu04216).

### Identification of differentially expressed genes (DEGs)

We used the “limma” package in R (4.1.1) to detect DEGs between the control and ALI groups [16]. The pheatmap package in R was used to visualize the differential gene expression heatmap (https://CRAN.R-project.org/package=pheatmap). A Venn plot was then constructed to show overlapping genes.

### Functional annotation analysis

The screened DEGs were evaluated using the “clusterProfiler” package in R to perform the Gene Ontology functional enrichment method [17]. Additionally, to analyze gene function, KEGG enrichment was performed using the “clusterProfiler” package in R [18]. Pathways with adjusted *p* < 0.05 were considered significantly enriched.

### Identification of characteristic genes

We used two methods of machine learning to further filter the target genes. LASSO regression is a compressed estimation method that obtains a more refined model by constructing a penalty function [19]. To screen out valuable genes, “glmnet” package in R was used for minimum absolute contraction and LASSO regression. A classification-supervised machine learning technique, called support vector machine (SVM) algorithm, trains subgroups with labels to condense its own set and identify the features that are most predictive [20]. In the present study, the predictive genes were obtained separately by LASSO regression and SVM algorithm, and the most potential characteristic genes were selected using the intersection set.

### Immunity analysis and gene expression

The CIBERSORT algorithm (https://cibersort.stanford.edu/) [21], a widely used technique for calculating the relative abundance of immune infiltrates, was used to determine the relative proportions of the 22 immune cells in the GSE2411 dataset.

### Gene set enrichment (GSEA)

We performed GSEA to further analyze the potential mechanisms and pathways of genes. The following thresholds of significance were employed: absolute NES values > 1, adjusted *p* values < 0.05, and FDR q-values = 0.25.

### Human BALF and blood analysis

Participants included in this study were enrolled between November 2022 and January 2023. ARDS was defined according to the Berlin Definition [22]. The studies involving human participants were reviewed and approved by Biomedical Research Ethics Committee, West China Hospital, Sichuan University (2019-337). After informed consent, BALF was collected within 24 h of the onset of ARDS. Blood samples were collected for laboratory data on the same day that the patient's BALF was collected. Clinical data including demographics, admission diagnosis, ARDS risk factors, laboratory data, APACHEII score, SOFA and outcomes were recorded. The expression of Cp (MU30288, Bioswamp, China), Slc7a11(MU13303, Bioswamp, China), Slc39a14 (MU13310, Bioswamp, China) and Gpx4 (MU31130, Bioswamp, China) in the patient’s BALF was determined using the ELISA kits.

### LPS-induced acute lung injury model

The experiment was approved by the Ethics Committee of West China Hospital of Sichuan University (20220609006). In the present study, 8- to 10-week-old male C57BL/6 mice were administered 2.5 μg/mL lipopolysaccharide (*Escherichia coli* serotype 0111: B4) via the trachea. After LPS administration for 6, 12, 24 and 48 h, the mice were euthanized and exsanguinated by cardiac puncture, and the lung tissue was removed for subsequent analysis.

### Isolation of primary neutrophils

Apical arterial blood was drawn from ALI/ARDS mouse model after LPS administration 24 h. Subsequently, neutrophils were isolated using mouse peripheral blood neutrophil isolation kit (LZS1100, TBD, China), as Additional file 2: Methods described, and resuspended in RIPM 1640 medium. Purity and viability of neutrophils were evaluated by flow cytometry.

### Cell culture and intervention

Cell culture of mouse lung epithelial cells (MLE-12) was purchased from ATCC (American Type Culture Collection, Manassas, USA). MLE-12 were cultured in a 37 °C humidified cell incubator with 5% CO_2_. DMEM medium (Gibco, USA) was chosen and supplemented with 10% fetal bovine serum (Gibco, USA). Twelve-well transwell culture plates (0.4 μm, Corning, USA) were used for the co-culture. MLE-12 cells were cultured in the lower chambers with DMEM medium, and primary neutrophils were cultured in the upper chambers with 1640 medium, for 24 h. Then, the co-cultured MLE-12 cells were collected for the next experiment. Additionally, the MLE-12 cells were treated with drug interventions, including Erastin (10 μM, Sigma-Aldrich, USA) and Ferr-1 (10 μM, Sigma-Aldrich, USA), depending on the experimental needs.

### Ferroptosis-related markers

To determine the occurrence of ferroptosis, we measured levels of iron, the lipid peroxidation metabolite malondialdehyde (MDA) and glutathione (GSH), by using tissue and cellular ferrous iron assay kits (E-BC-K773-M and E-BC-K881-M, Elabscience, China), MDA assay kits (E-BC-K025-M and E-BC-K028-M, Elabscience, China) and GSH assay kits (RK05819, ABclonal, China). Mitochondrial membrane potential was measured using JC-1 mitochondrial membrane potential assay dye (E-CK-A301, Elabscience, China), following the manufacturer's instructions. The ultrastructural changes of ferroptosis in lung tissue were examined by transmission electron microscopy (Hitachi H-7800, Hitachi, Naka, Japan), which was performed by Hubei BIOSSCI Biotech Co., Ltd.

### Immunofluorescence, qRT-PCR and Western blot

The assays for immunofluorescence (IF), quantitative real-time PCR (Additional file 2: Table S1) and Western blot (Additional file 2: Table S2) were performed as described in Additional file 2: Methods. Full Western blot images are shown in Additional file 1.

### Statistical analysis

In the present study, R software (version 3.6.3) and GraphPad Prism (version 9.0) were used for all statistical analyses. Differentially expressed genes between the two groups were identified using Fisher’s exact test. Correlations between genes and immune cells and co-location analysis were analyzed using Pearson's method. LASSO regression analysis and SVM were performed using R packages. Intensity and co-localisation of immunofluorescence for quantitative analysis using ImageJ (version 2.0.0). For qRT-PCR, Western blot, Elisa, comparisons between groups were performed using one-way ANOVA. Statistical significance was defined as *p* < 0.05.

## Results

### DEG screening and functional enrichment analysis

**Fig. 1:**
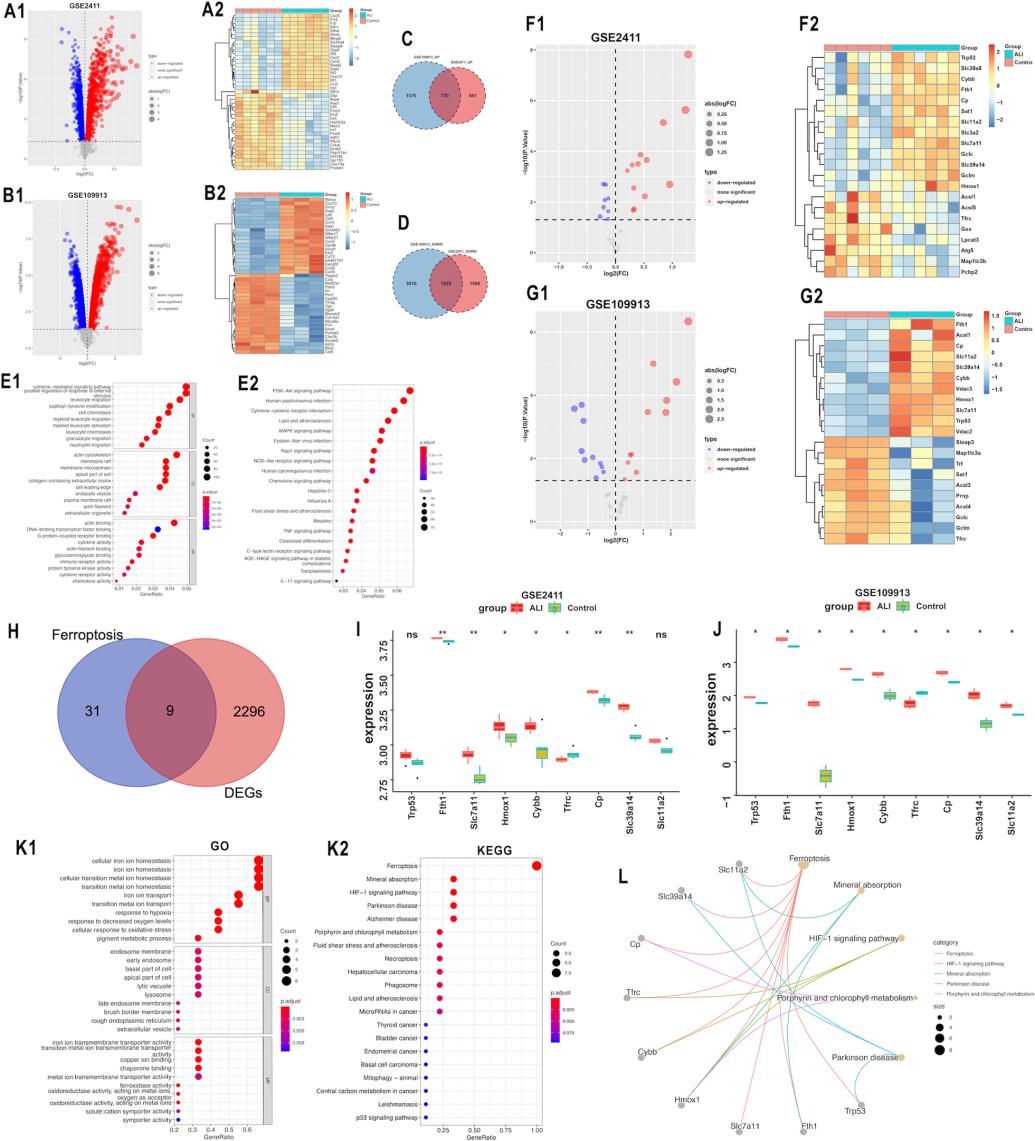
Identification of candidate FRGs. **A1** Volcano map of DEGs between acute lung injury group and control group in GSE2411 dataset. **A2** Heatmap of DEGs from GSE2411 dataset. **B1** Volcano map of DEGs between acute lung injury group and control group in GSE109913 dataset. **B2** Heatmap of DEGs from GSE109913 dataset. **C** Venn plot of upregulated overlapping genes in two datasets. **D** Venn plot of downregulated overlapping genes in two datasets. **E1** GO enrichment analysis of DEGs. **E2** KEGG pathway enrichment analysis of DEGs. **F1** Volcano map of FRGs between acute lung injury group and control group in GSE2411 dataset. **F2** Heatmap of FRGs from GSE2411 dataset. **G1** Volcano map of FRGs between acute lung injury group and control group in GSE109913 dataset. **G2** Heatmap of FRGs from GSE109913 dataset. **H** Venn plot for the overlapping genes between FRGs and DEGs. **I** The expression level of the nine candidate FRGs in the ALI group and control group of GSE2411 dataset. **J** The expression level of the nine candidate FRGs in the ALI group and control group of GSE109913 dataset. **K1** GO enrichment analysis of the nine candidate FRGs. **K2** KEGG pathway enrichment analysis of the nine candidate FRGs. **L** Correlation of top 5 KEGG results with the nine candidate FRGs. ns, not significant; *p < 0.05; **p < 0.01

Under the criteria of adjusted *p* < 0.05 and absolute log2-fold-change (FC) > 0, the two GSE109913 and GSE2411 datasets were screened for differential genes (Fig. 1A, B). The intersection of the up- and downregulated DEGs from each of the two datasets was then calculated, which identified 770 overlapping upregulated DEGs (Fig. 1C) and 1535 overlapping downregulated DEGs (Fig. 1D). All overlapping DEGs were functionally enriched, and the significant GO keywords and KEGG pathway are presented in the Fig. 1E.

### Identification of candidate ferroptosis-related genes (FRGs)

Further, we obtained mouse FRGs from the KEGG database, and Additional file 4: Table S3 provides a list of them. The FRGs in GSE2411 and GSE109913 were then abstracted (Fig. 1F, G, Additional file 4: Tables S4, S5). Nine overlapping genes were subsequently detected after utilizing a Venn diagram to overlap FRGs and DEGs, namely, Trp53, Fth1, Slc7a11, Hmox1, Cybb, Tfrc, Cp, Slc39a14 and Slc11a2 (Fig. 1H). The specific expression of the nine candidate FRGs in the two datasets are shown in Fig. 1I, J. We utilized GO and KEGG analysis to determine the function of these nine candidate FRGs in relation to ALI/ARDS (Fig. 1K, L). KEGG suggested that in ALI/ARDS, these nine candidate FRGs were associated not only with ferroptosis but also with the HIF-1 signaling pathway, which are closely related to the acute hypoxic response in ALI/ARDS.

### Further screening of characteristic genes with machine learning

**Fig. 2:**
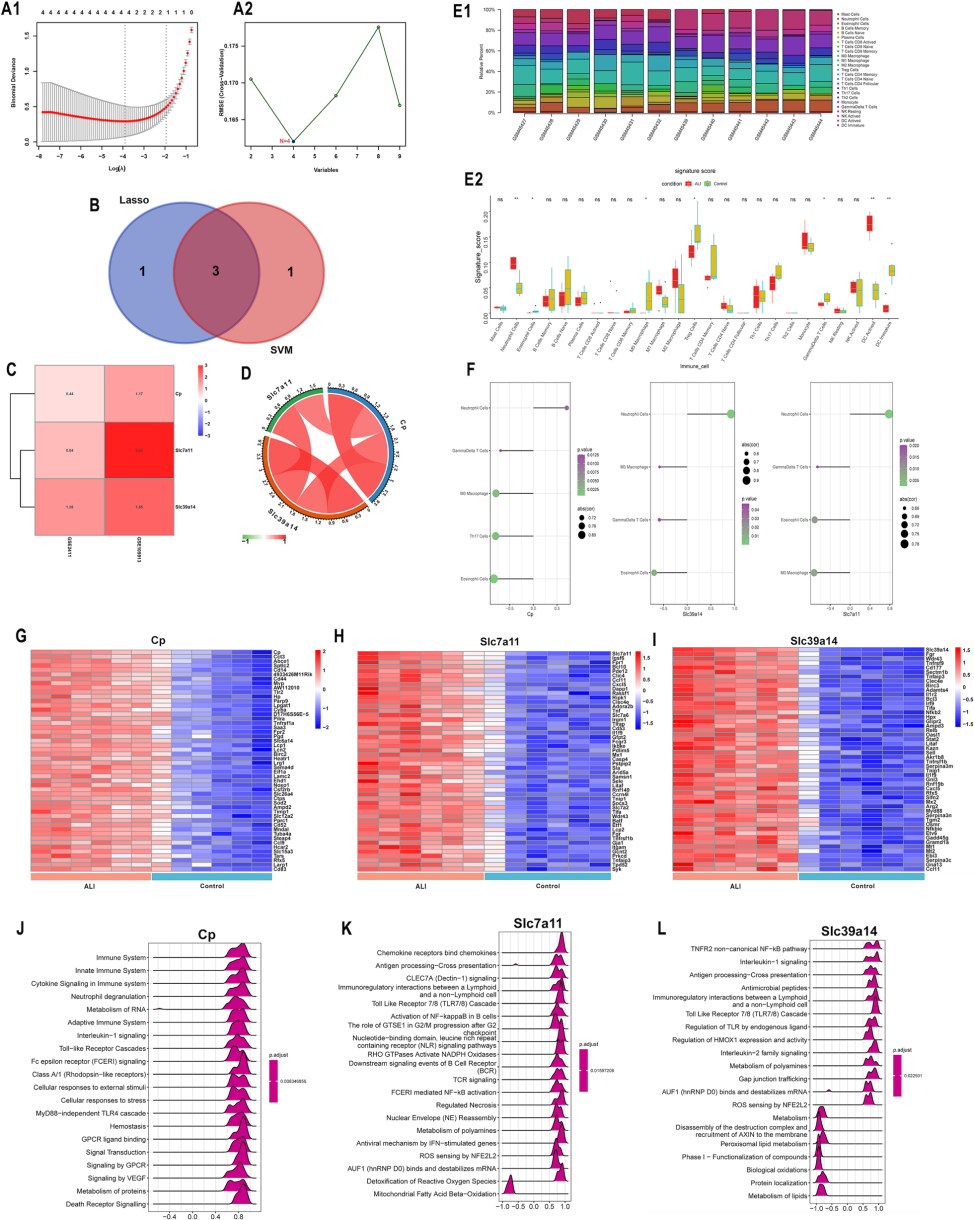
Identification of characteristic FRGs and immune infiltration analysis. **A1** Lasso regression and **A2** SVM analysis. **B** Venn plot for the overlapping genes between the results of Lasso regression and SVM analysis. **C** Heatmap of the three characteristic genes in GSE2411 and GSE109913. **D** The correlation among the three characteristic genes. **E1** Immune landscape in ALI (GSM45427–GSM45432) and control (GSM45439–GSM45444) samples based on CIBERSORT analysis in GSE2411 datasets. **E2** The distribution patterns of 22 immune cells between the ALI and control group in GSE2411 datasets. **F** The correlation of three core genes with immune cell infiltration (*p* < 0.05). **G** Heatmap of correlation analysis of Cp and its related up-regulated genes. **H** Heat map of correlation analysis of Slc7a11 and its related up-regulated genes. **I** Heatmap of correlation analysis of Slc39a14 and its related up-regulated genes. **J** The GSEA of top 20 related functions based on the correlation analysis of Cp. **K** The GSEA of top 20 related functions based on the correlation analysis of Slc7a11. **L** The GSEA of top 20 related functions based on the correlation analysis of Slc39a14. ns, not significant; *p < 0.05. **p < 0.01

To further narrow the scope to screen for characteristic FRGs in ALI/ARDS, LASSO regression and SVM analysis were performed to identify characteristic genes. The protein–protein network (PPI) is shown in Additional file 3: Fig. S1. As a result, four out of the nine FRGs, namely, Slc7a11, Tfrc, Cp, and Slc39a14, were selected after LASSO regression analysis (Fig. 2A1). Four out of the nine FRGs, namely, Slc39a14, Slc7a11, Cp, and Fth1, were selected after SVM regression analysis (Fig. 2A2). We then identified the intersection of the genes. And finally, three genes, namely, Cp, Slc7a11 and Slc39a14, were identified as characteristic genes highly associated with ALI/ARDS (Fig. 2B–D). We also further validated the expression of the three genes in GSE17355 dataset (Additional file 3: Fig. S2).

### Immune annotation

To further explored the immune pattern of ALI/ARDS, CIBERSORT analysis was performed to characterize the immune infiltration in ALI and to evaluate the composition of immune cells (Fig. 2E). The immune cells infiltrating the lungs varied in the ALI-treated (GSM45427–GSM45432) and control groups (GSM45439–GSM45444) with significant enrichment of neutrophils (*p* < 0.01) and activated dendritic cells (*p* < 0.01) in the ALI group. There was a significant decrease in M0 macrophages (*p* < 0.05) in the ALI group and a corresponding increase in M1 and M2 macrophages, and there the increase in M2 macrophages was predominant. The levels of Tregs (*p* < 0.05), gamma delta T cells (*p* < 0.05) and immature dendritic cells (*p* < 0.01) in the ALI group were lower than those in the control group, which was consistent with the inflammatory activation characteristic of ALI, and there was no trend toward immunosuppression.

Correlation analysis showed that expression of Cp, Slc7a11 and Slc39a14 were associated with increased infiltration of neutrophils but negatively associated with infiltration of immature dendritic, gamma delta T cells, eosinophils and M0 macrophages. In addition, positive expression of the Cp gene was also associated with suppressed expression in Th17 cells (Fig. 2F, Additional file 4: Tables S6–S8).

### Functional annotation of GSEA

Reactome-based GSEA of a single gene was performed to further elucidate the mechanism of the upregulated genes (Fig. 2G–L). In addition to being associated with the innate and adaptive immune systems, Slc7A11 gene was also associated with the inhibition of mitochondrial fatty acid beta-oxidation. Slc39a14 was associated with activation of the TNFR2 noncanonical NF-KB pathway, interleukin-1 signaling pathway, recruitment of AXIN to the membrane, peroxisomal lipid metabolism and other pathways.

### Validation of the expression levels of selected characteristic FRGs in human BALF

A prospective observational study of ARDS patients was conducted to validate the concentrations of characteristic FRGs in BALF sample. The clinical characteristics of patients are listed in Table 1. The study population included 59 participants with ARDS and were classified according to the Berlin definition as mild, moderate and severe. The APACHEII score for the total study population was 20.0 (7.5), the total length of stay was 28.2 (19.6) days with the ICU mortality as 40.7%.

**Table 1:** Baseline patient characteristics

	Total (n = 59)	Mild ARDS (n = 11)	Moderate ARDS (n = 29)	Severe ARDS (n = 19)	*p* value
Age, yr	59.2 (15.6)	55.3 (13.5)	58.6 (16.5)	62.0 (15.7)	0.53
Sex, male, %	40 (67.8%)	6 (54.5%)	18 (62.1%)	15 (78.9%)	0.28
BMI	24.6 (3.2)	23.7 (3.0)	24.0 (2.9)	26.1 (3.3)	0.06
Leukocytes, %	12.7 (6.7)	12.3 (4.1)	12.1 (6.5)	13.9 (8.3)	0.68
Neutrophils, %	85.3 (7.6)	85.4 (3.1)	84.4 (9.8)	87.0 (5.1)	0.55
CRP, mg/L	137.0 (104.9)	148.2 (118.0)	115.0 (90.0)	163.2 (115.8)	0.30
IL-6, ng/L	433.0 (915.9)	157.6 (178.8)	275.6 (540.9)	837.3 (1409.3)	0.07
PCT, ng/mL	16.5 (27.9)	4.2 (7.6)	16.9 (31.1)	23.4 (29.2)	0.20
PaO_2_/FiO_2_ ratio	155.4 (83.9)	284.2 (60.5)	148.3 (29.0)	73.3 (18.8)	<0.01*
Admission diagnosis, n (%)					
Trauma	13 (22.0%)	2 (18.2%)	8 (27.6%)	3 (15.8%)	0.56
Sepsis	12 (20.3%)	1 (9.1%)	7 (63.6%)	4 (21.1%)	0.51
Pneumonia	10 (16.9%)	3 (27.3%)	5 (17.2%)	2 (10.5%)	0.57
Respiratory failure	5 (8.5%)	1 (9.1%)	1 (3.4%)	3 (15.8%)	0.32
Intracranial hemorrhage	8 (13.6%)	4 (36.4%)	3 (10.3%)	1 (5.3%)	0.07
Severe pancreatitis	11 (18.6%)	0 (0.0%)	5 (17.2%)	6 (31.5%)	0.16
ARDS risk factor, n (%)					0.44
Direct^a^	25 (42.3%)	6 (54.5%)	13 (44.8%)	6 (31.6%)	
Indirect^b^	34 (57.6%)	5 (45.5%)	16 (55.2%)	13 (68.4%)	
APACHE II	20.0 (7.5)	15.0 (5.7)	19.6 (6.7)	23.5 (8.1)	0.02*
SOFA	11.5 (4.1)	8.9 (2.3)	11.3 (4,2)	13.4 (3.9)	0.03*
ICU duration, days	28.2 (19.6)	32.5 (15.0)	30.7 (23.7)	21.7 (12.6)	0.23
Death in ICU, %	24 (40.7%)	1 (9.1%)	12 (41.4%)	11 (57.9%)	0.02*

Data presented as mean (standard deviation) and count(percentage)

*p < 0.05 was statistically significant

*APACHE II* Acute Physiology and Chronic Health Evaluation II, *BMI* body mass index, *SOFA* sepsis-related organ failure assessment

The* p* value compared between the three groups of mild, moderate, and severe ARDS were performed using one-way ANOVA

^a^Direct ARDS was defined as pneumonia, aspiration, inhalation injury, near drowning, or lung contusion as the risk factor for ARDS

^b^Indirect ARDS was defined as extrapulmonary sepsis or nonthoracic trauma as the risk factor for ARDS

**Fig. 3:**
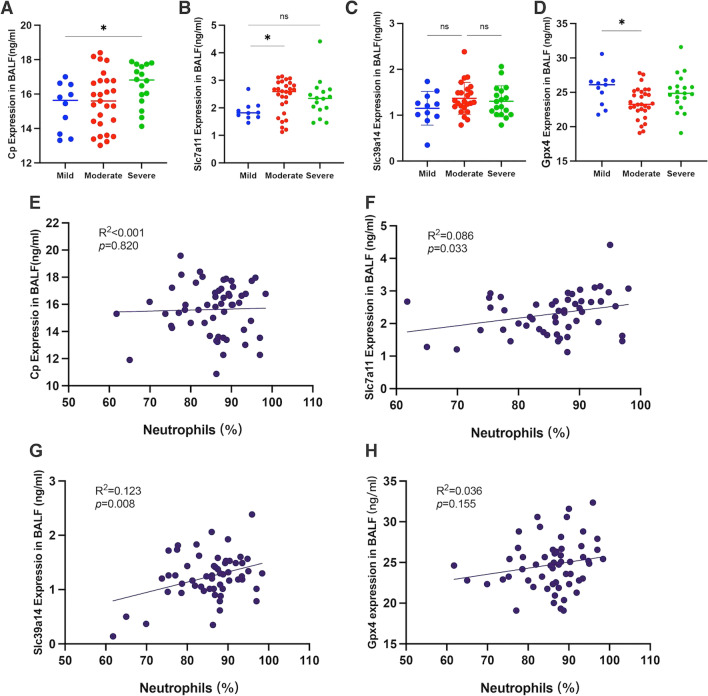
Concentrations of characteristic FRGs in BALF from patients with acute respiratory distress syndrome (Mild ARDS, n = 11; Moderate ARDS, n = 29; Severe ARDS, n = 19). **A** Cp expression in BALF; **B** Slc7a11 expression in BALF; **C** Slc39a14 expression in BALF; **D** Gpx4 expression in BALF; **E** correlation analysis between the expression of Cp in BALF and the percentage of peripheral blood neutrophils; **F** correlation analysis between the expression of Slc7a11 in BALF and the percentage of peripheral blood neutrophils; **G** correlation analysis between the expression of Slc39a14 in BALF and the percentage of peripheral blood neutrophils; **D** correlation analysis between the expression of Gpx4 in BALF and the percentage of peripheral blood neutrophils; Pearman R^2^ and* p* values were computed as indicated on each graph

We collected BALF from clinical ARDS patients to verify the expression of Cp, Slc7a11 and Slc39a14 in humans. The results showed that in the BALF of ARDS patients, Cp was significantly elevated in patients with severe ARDS (*p* = 0.019), Slc7a11 was significantly elevated in patients with moderate ARDS (*p* = 0.021), whereas Slc39a14 was not significantly elevated, relative to patients with mild ARDS (Fig. 3). Gpx4 is the core enzyme that regulates the antioxidant system (glutathione system) and its reduction is an important marker of ferroptosis. In our study, there was a significant decrease in Gpx4 expression levels in moderate ARDS (*p* = 0.049).

By exploring the relationship between characteristic FRGs and the immune status of patients, we found that the levels of neutrophils in the peripheral blood of ARDS patients were positively correlated with the expression levels of Slc7a11 (Pearson’s R^2^ = 0.086,* p* = 0.033) and Slc39a14 (Pearson’s R^2^ = 0.123, *p* = 0.008). CD3 and CD8 are markers specific to the surface of T lymphocytes, and an increase in Slc7a11 correlates with a decrease in peripheral blood CD3 (*p* = 0.043) and CD8 (*p* = 0.018) levels, possibly indicating suppression of the T cell immune response (Additional file 3: Fig. S3 and Additional file 4: Table S9).

### Ferroptosis was rapidly activated in the ALI/ARDS mouse model

**Fig. 4:**
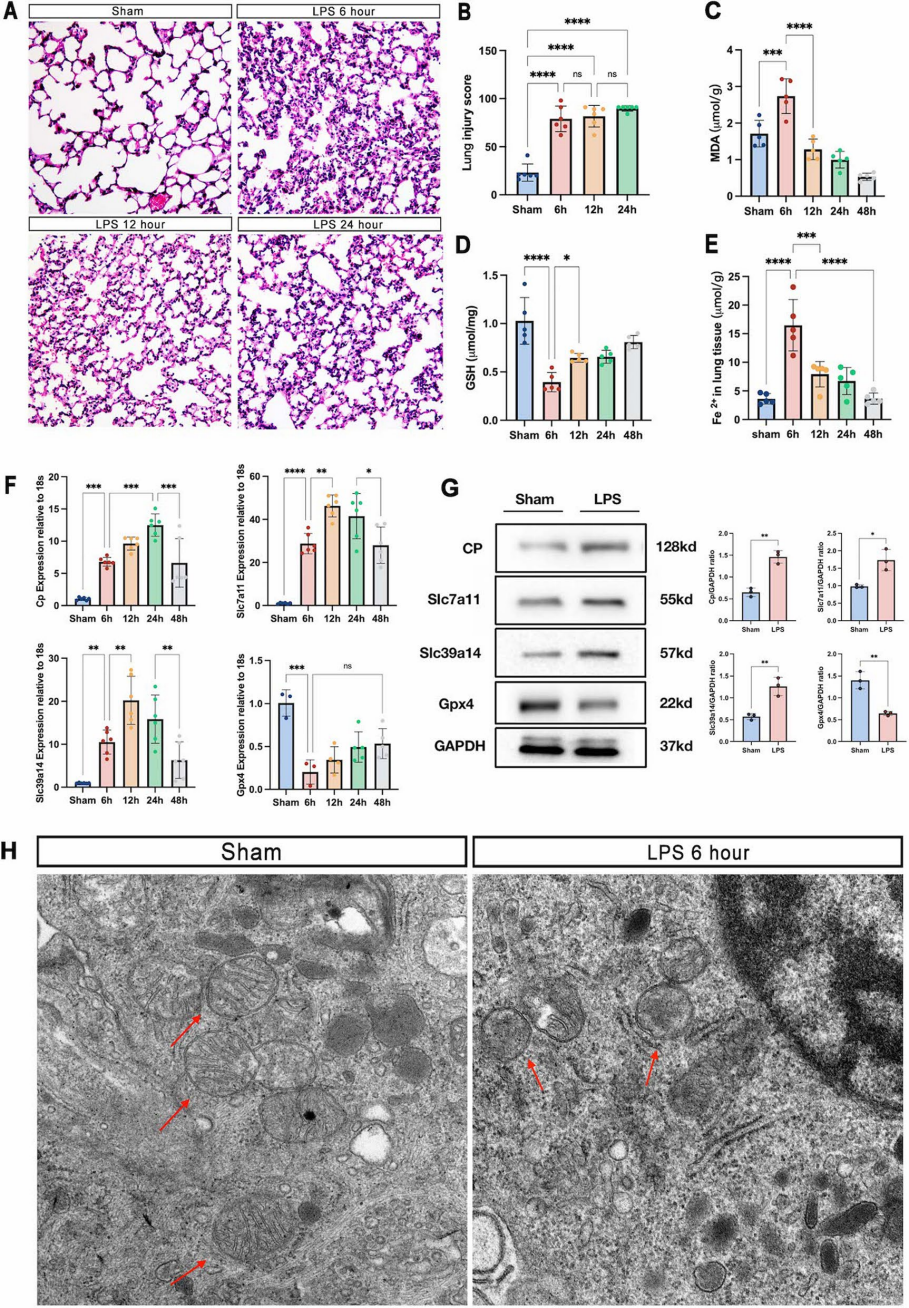
LPS administration induces lung injury and ferroptosis. **A** The representative H&E staining of lung tissue sections, magnification ×400; **B** the lung injury score analysis; the level of ferroptosis markers, **C** MDA, **D** GSH, **E** Fe^2+^ in lung tissue; **F** the mRNA levels of Cp, Slc7a11, Slc39a14 and Gpx4 in lung tissue were detected by real-time qPCR. **G** The Cp, Slc7a11, Slc39a14 and Gpx4 protein levels in lung tissue were detected by western blotting at 6 h; **H** Ultrastructural changes of mitochondria in lung tissue by transmission electron microscopy, magnification ×100,000. Data presented as mean ± standard deviation with *p < 0.05, **p < 0.01, ***p < 0.001, ****p < 0.0001 for all statistics

We established a model of LPS-induced ALI/ARDS and significant lung injury was observed in lung tissue for 6 h after modelling (Fig. 4A, B). We further examined markers of ferroptosis in lung tissue, including MDA content (Fig. 4C), levels of the GSH (Fig. 4D), and iron accumulation (Fig. 4E). We found that after LPS administration, lung tissue produced a large amount of MDA, a lipid oxidation product, in a short period of time (6 h), accompanied by a large depletion of GSH and the accumulation of iron, which was consistent with ferroptosis characterized by intense and rapid lipid oxidative stress. This response was slowly compensated within 12 to 48 h and the markers of ferroptosis gradually returned to normal levels. Meanwhile, Gpx4, a marker gene for ferroptosis, was significantly reduced at 6 h and progressively upregulated at 12 to 48 h.

Ferroptosis is characterized by the accumulation of lipid peroxides, resulting in the breakdown of cell membranes and ultimately, mitochondrial dysfunction [23]. This dysfunction is caused by a decrease in the production of ATP and the release of cytochrome c, leading to oxidative stress and damage to the mitochondrial DNA. Electron microscopy and membrane potential assays are commonly used to assess the extent of ferroptosis mitochondrial damage [24, 25]. Electron microscopy revealed 6 h after the administration of LPS-induced lung injury, a shrinkage of intracellular mitochondria, a thickening of the density of mitochondrial bilayer membrane structures and a reduction in internal mitochondrial cristae, consistent with the mitochondrial damage characteristic of ferroptosis (Fig. 4H).

After ALI/ARDS modeling, the expression of characteristic genes Cp, Slc7a11 and Slc39a14 were progressively upregulated and peaked at 12 to 24 h (Fig. 4F, G). The results above showed that the three characteristic genes were significantly activated after the onset of ferroptosis (6 h) early in ALI/ARDS, and that ferroptosis was alleviated after the organism compensated within 12 to 48 h.

By immunofluorescence staining, we observed the relationship between neutrophil infiltration and Slc7a11 expression.

**Fig. 5:**
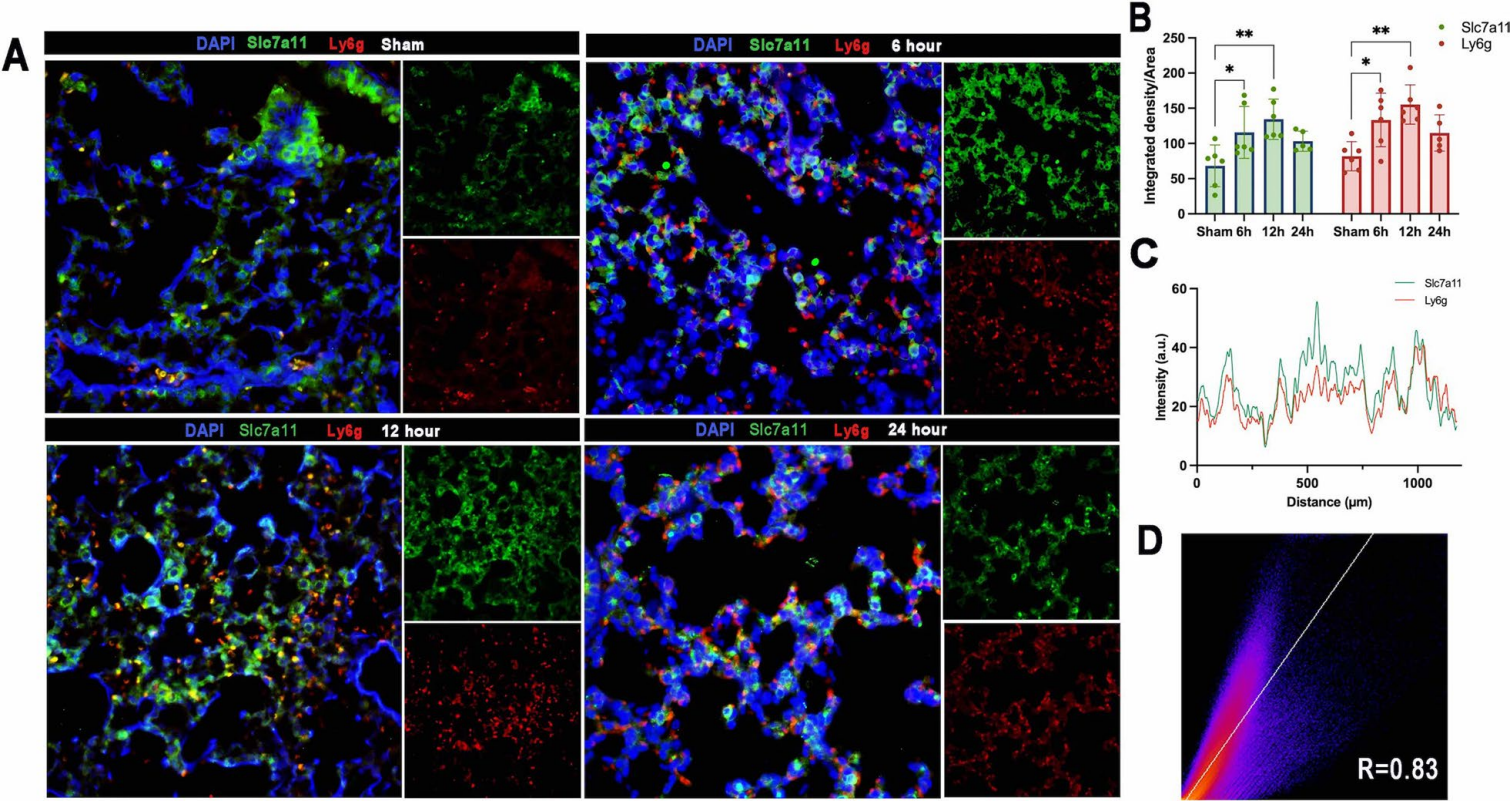
**A** Representative immunofluorescence staining for Slc7a11 (green) and Ly6g (red) in lung tissue at 6, 12, 24 h, magnification ×400; **B** bar graph of fluorescence intensity of Slc7a11 and Ly6g; **C**, **D** Immunofluorescence co-localisation analysis of Slc7a11 and Ly6g in 12 h; Data presented as mean ± standard deviation with *p < 0.05, **p < 0.01, ***p < 0.001, ****p < 0.0001 for all statistics; Pearman R values were computed as indicated on graph

The findings demonstrated that 6 h after lung injury, there was a considerable recruitment of neutrophils, which peaked at 12 h (Fig. 5A, B). Slc7a11 is mostly expressed in alveolar extracellular matrix. As neutrophils were drawn into the lung in huge numbers, Slc7a11 expression was also noticeably elevated. Interestingly, at 12 h, Slc7a11 and neutrophil co-localization in the alveolar was considerable (Fig. 5C, D). Slc7a11’s expression profile region mainly overlapped neutrophils, and Pearson correlation analysis found a strong association (R = 0.83) between the two. These data suggested that the recruitment of activated neutrophils from the periphery to the lung after injury appears to be associated with an upregulation of Slc7a11.

### Neutrophils recruited to the lungs compensate ferroptosis after ALI/ARDS

**Fig. 6:**
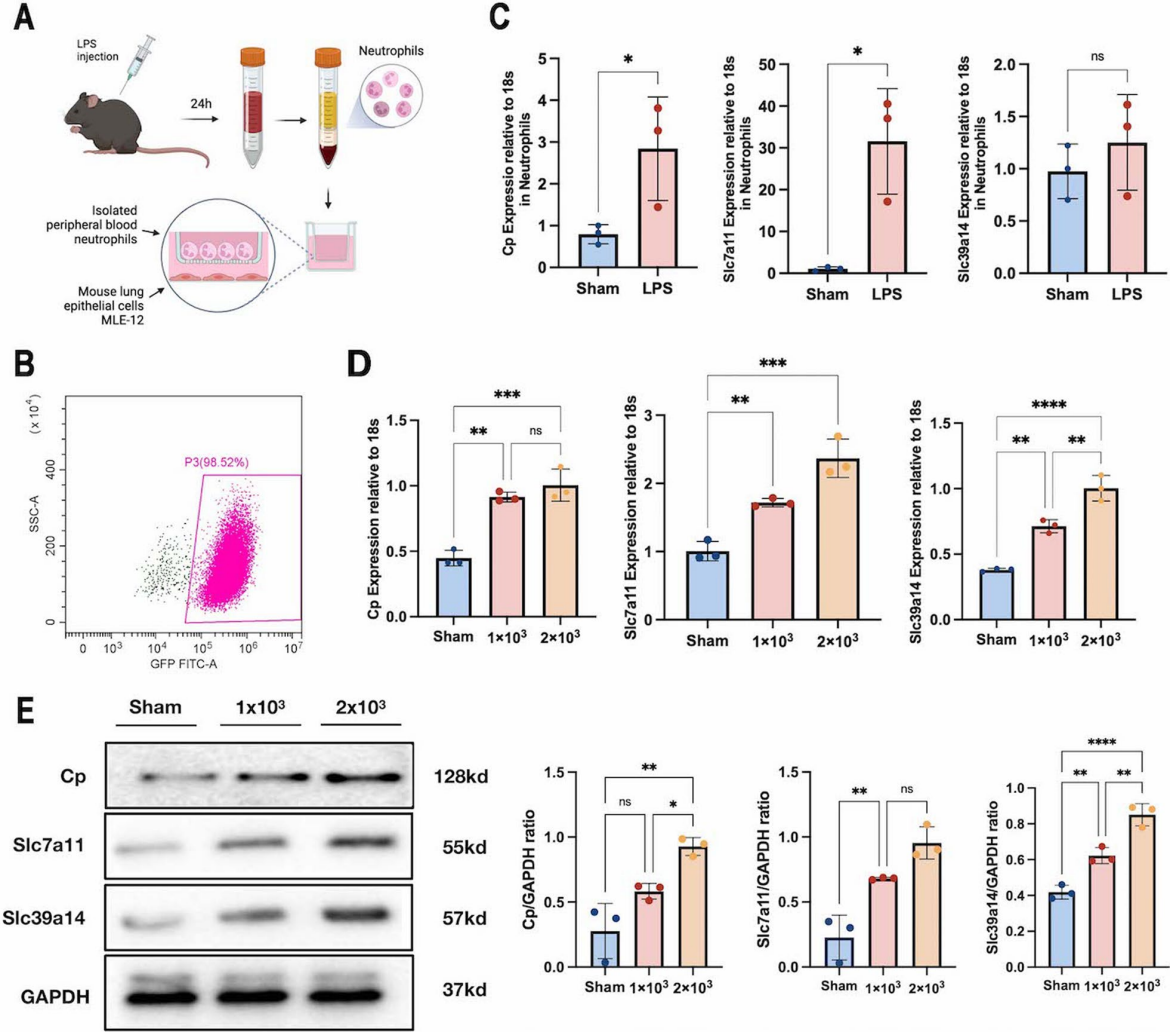
**A** Illustration of isolation of peripheral blood neutrophils and co-culture; **B** verification of the purity of isolated neutrophils by flow cytometry; **C** mRNA expression of Cp, Slc7a11 and Slc39a14 in isolated neutrophils; **D** mRNA expression of Cp, Slc7a11 and Slc39a14 in MLE-12 co-cultured with different numbers of neutrophils; **E** protein levels of Cp, Slc7a11 and Slc39a14 in MLE-12 co-cultured with different numbers of neutrophils; data presented as mean ± standard deviation with *p < 0.05, **p < 0.01, ***p < 0.001, ****p < 0.0001 for all statistics

To test the hypothesis, we isolated primary activated neutrophils from mice and co-cultured them with lung epithelial cells (MLE-12) in transwell, to mimic the effect of neutrophils recruited to the lung during ALI/ARDS (Fig. 6A). In the upper chambers, Cp and Slc7A11 were highly elevated in active neutrophils, but Slc39a14 was not significantly upregulated, according to qRT-PCR results on isolated activated neutrophils (Fig. 6C). In the lower chambers, Slc7a11, Cp and Slc39a14 in MLE-12 cells were significantly upregulated as the number of neutrophils increased (Fig. 6D). This phenomenon implied that a neutrophil derived factor upregulated the characteristic FRGs in the lung epithelium.

**Fig. 7:**
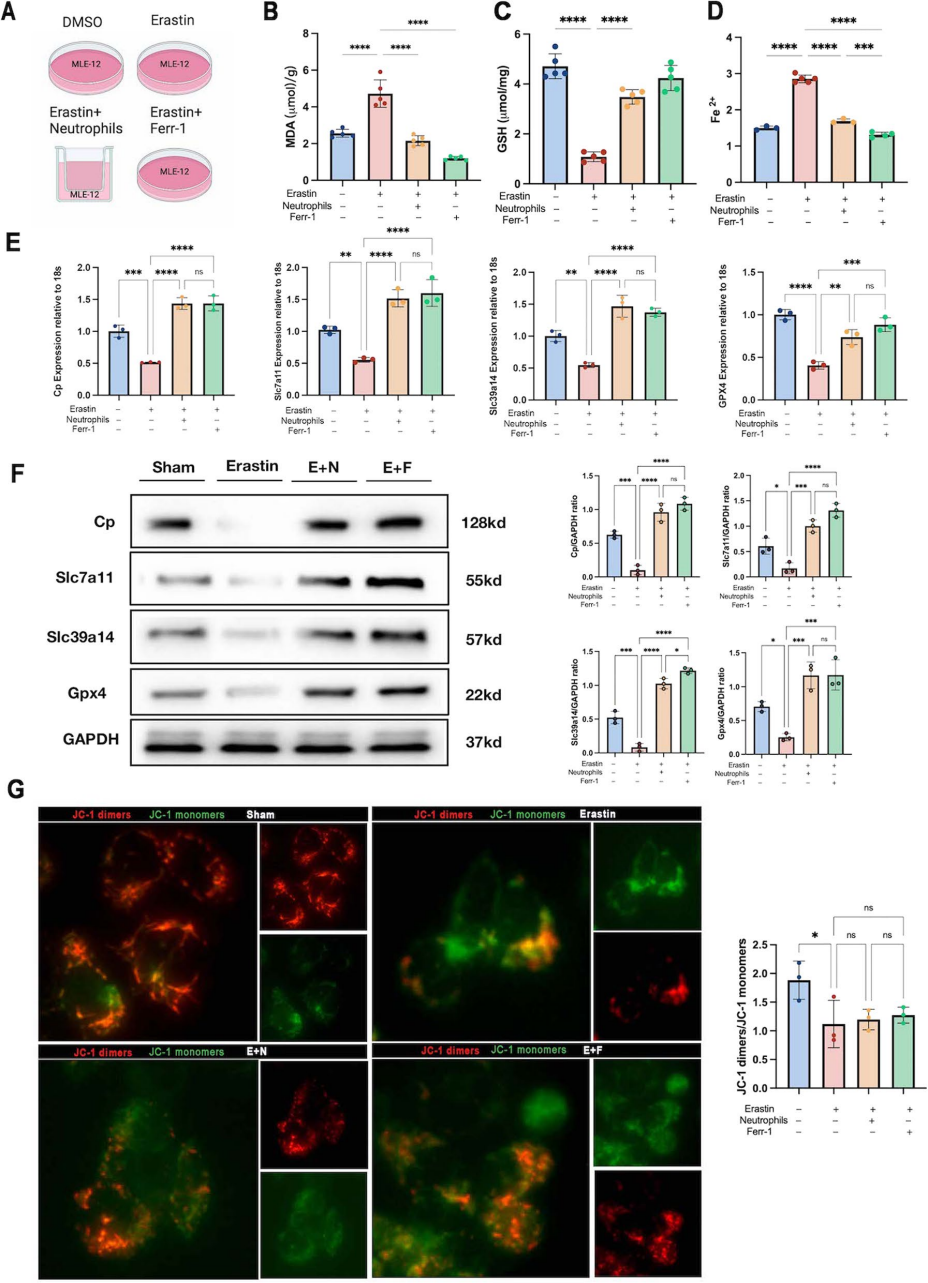
**A** Diagram of the different groups of drug administration; the level of ferroptosis markers **B** MDA, **C** GSH, **D** Fe^2+^ in MLE-12 cell; **E** the mRNA levels of Cp, Slc7a11, Slc39a14 and Gpx4 in MLE-12 cell; **F** the Cp, Slc7a11, Slc39a14 and Gpx4 protein levels in MLE-12 cell; **G** fluorescent expression of the mitochondrial membrane potential (JC-1) with bar graph of fluorescence intensity of JC-1 dimers (red) and JC-1 monomers (green), magnification ×400; data presented as mean ± standard deviation with *p < 0.05, **p < 0.01, ***p < 0.001, ****p < 0.0001 for all statistics

Further, to understand for what purpose neutrophils regulate the characteristic FRGs elevation, we investigated if this effect had an impact on lipid peroxidation produced by ferroptosis (Fig. 7A).

Erastin is a ferroptosis inducer which inhibits the Slc7a11 channel protein, thereby blocking the Slc7a11-GSH/Gpx4 axis and inducing ferroptosis. The lipid oxidative stress generated during ferroptosis will disrupt the integrity of the inner mitochondrial membrane, leading to a decrease in mitochondrial membrane potential [23]. Reduced membrane potential in JC-1 assay as a shift from JC-1 dimers (red) to JC-1 monomers (green), the reduced red/green fluorescence intensity ratio representing mitochondrial dysfunction. After administration of erastin, significant MDA, ferrous iron was produced, along with a significant reduction in GSH (Fig. 7B–D) and mitochondrial membrane potential (Fig. 7G). Slc7a11 and Gpx4 levels decreased when the Slc7a11-GSH/Gpx4 axis was inhibited, both at the gene and protein levels (Fig. 7E, F).

Ferr-1 is one of the current inhibitors of ferroptosis and can effectively inhibit the oxidative stress products produced by ferroptosis. After administration of ferr-1, ferroptosis was effectively reversed accompanied by a rise in Slc7a11 and Gpx4 (Fig. 7E, F). To verify the effect of peripheral blood neutrophils on ferroptosis, we established a co-culture system in which primary peripheral blood neutrophils were given co-culture after erastin induced ferroptosis in lung epithelial cells.

The results showed that neutrophil infiltration alleviated erastin-induced MDA accumulation, GSH depletion, and iron accumulation, accompanied by upregulation of Slc7a11 and Gpx4. The alleviating ferroptosis effect of neutrophils has a similar role to that of Ferr-1, implying the existence of a compensatory effect of lipid oxidation in neutrophils after acute lung injury in the organism. However, neither neutrophils nor Ferr-1 failed to increase mitochondrial membrane potential (Fig. 7G). This phenomenon is consistent with previous research and may be attributed to the limited compensatory effects of neutrophils or Ferr-1, as well as the presence of alternative pathways regulating mitochondrial damage [26, 27].

## Discussion

Although several studies have demonstrated the role of genes associated with ferroptosis in the onset and progression of ALI, their immune infiltration pattern, and immune-regulated interactions with ferroptosis in ALI are still ambiguous. In the present study, we identified three characteristic FRGs, Cp, Slc39a14 and Slc7a11, by analyzing two gene expression profiling datasets. Immune infiltration analysis showed that the three characteristic FRGs were significantly positively correlated with the infiltration levels of neutrophils. We further prospectively collecting BALF from 59 ARDS patients and found that Slc7a11 was significantly increased in the BALF of patients with moderate ARDS and had a positive linear relationship with peripheral blood neutrophil levels. Based on LPS-induced ALI/ARDS model, we found that infiltration of peripheral blood neutrophils into the lung could compensate for ferroptosis occurring in early onset lung injury, implying the existence of a compensatory effect of lipid oxidation in peripheral blood neutrophils in the organism.

Among ferroptosis-related genes, the decreases in Slc7a11 and Gpx4 are markers of ferroptosis [28, 29]. However, in our study, both clinical samples and animal ALI/ARDS models showed upregulation of Slc7a11 expression during acute lung injury, which might be regulated by peripherally recruited neutrophils to the lung, suggesting a different regulation of ferroptosis-related genes than previously thought. The cystine transporter system Xc− component, Slc7a11, is essential for maintaining intracellular GSH levels as well as the exchange and balance of glutamate [30]. GSH is essential for maintaining cellular homeostasis, as GSH deficiency triggers oxidative stress and immune imbalance in the body [31]. One recent study found that Slc7a11 can influence the intracellular (and extracellular) redox imbalance in T2-high asthma by regulating the GSH/GSSG ratio [32]. And another study found that activated neutrophils affect intracellular GSH levels, and that FALD-GSH levels may be produced at the site of neutrophil activation [33]. And as an important protein channel for GSH synthesis, there is a high probability that Slc7a11 has an effect on it under the influence of activated neutrophils. This might explain why Slc7a11 displayed an increase trend triggered by neutrophils in our study. Although upregulation of Slc7a11 implies increased synthesis of GSH, high levels of Slc7a11 are still associated with high levels of inflammation in the organism [34]. A recent study has reported that Slc7a11 is upregulated in innate immune cells and that inhibition of Slc7a11 accelerates inflammatory wound healing [34]. Therefore, the increase in Slc7a11 may have a dual role of proinflammatory effects and anti-oxidative stress. In the present study, our hypothesis is that during ALI/ARDS, pulmonary Slc7a11 is constantly upregulated by peripherally recruited neutrophils to the lung, thereby compensating for the oxidative stress caused by lung injury, which may be part of a fine balance of signaling regulation in vivo. The increase in Slc7a11 in the organism following acute lung injury implies that repair mechanisms under inflammatory activation are compensating at the same time. However, although the purpose of this upregulation is meant to compensate, the consequences may leave the organism in a state of compensatory imbalance.

Slc39a14 (ZIP14) is a transmembrane transport protein with selective abilities for iron and zinc as well as a preference for transporting manganese [35]. In the presence of iron overload, Slc39a14 transports iron and mediates the development of ferroptosis, thereby affecting the development of various diseases, such as glucolipid metabolism disorders, liver fibrosis and intestinal dysfunction [36, 37]. Slc39a14 is mainly expressed in type II alveolar epithelium, fibroblasts, and endothelial cells in the lung. Although Slc39a14 expression is not high in the lung, a previous study using A549 epithelial cells has reported that it plays an important role in metabolism and metal transport [38]. Moreover, Slc39a14 is regulated by immunity and inflammation. A previous study has investigated the role and regulation of Slc39a14 in activating the macrophage inflammatory response, and it reported that Slc39a14 is continuously upregulated in activated macrophages [39]. This study found that either iron overload or iron depletion reduced Slc39a14 expression, however, LPS significantly stimulated the upregulation of Slc39a14, thereby preventing an excessive inflammatory response. This finding corroborates our results from another perspective that the upregulation of Slc39a14 after acute inflammation is associated with a compensatory effect of immune cells.

Ceruloplasmin, also known as Cp, is the main copper transport protein in plasma and lung epithelial cells [40]. Cp is an acute phase protein that lowers the production of reactive oxygen species mediated by Fe^2+^ during iron metabolism, thereby reducing the cellular damage caused by harmful peroxidation products [41]. Cp is widely regarded as a potent antioxidant stress shield. The overexpression of Cp has been found to suppress ferroptosis, and elevated Cp promotes HIF1α expression by reducing Fe^2+^, forming a positive feedback loop [42].

Although Cp is an antioxidant and is beneficial to iron homeostasis, it is associated with poor prognosis in a variety of diseases in a highly inflammatory state. High ceruloplasmin levels are associated with type I/II diabetes, metabolic syndrome and other recognized risk factors for cardiovascular disease [43].

Moreover, elevated levels of Cp have also been detected in BALF taken from patients with ARDS [44]. Similarly, in our study, Cp was elevated in BALF in patients with severe ARDS, while there was no significant difference in moderate ARDS patients. Despite the presence of neutrophil-induced upregulation of Cp expression in the LPS-induced ALI/ARDS model, no significant correlation was observed in the BALF of patients, possibly related to species differences. As a ferroptosis-related gene, however, the role of Cp in acute lung injury with an unstable internal immune environment is not clear, and it remains unknown whether Cp plays an antioxidant or a proinflammatory role.

Additionally, the present study had several limitations. Firstly, the patients included in this study met the clinical need for fibrotic bronchoscopy. As most ICU patients had varying degrees of lung injury, we were unable to collect BALF from patients without lung injury, as performing fibrotic bronchoscopy in this group of patients would instead be an impairment. Therefore, in this study, data could only be collected from patients with mild ARDS as a control, and inflammatory activation may already be present in the BALF of patients with mild ARDS. Secondly, the mechanisms of how neutrophils regulate genes and the purpose of their regulation still need to be further explored in gene target mice.

Although neutrophils exhibit a compensatory role in rescuing ferroptosis early in ALI model, excessive neutrophil infiltration is theoretically associated with poor outcomes in ALI/ARDS [45]. Therefore, whether neutrophil-induced Slc7a11 upregulation brings benefits or metabolic imbalance to the organism remains unclear, and the mysterious dual role of neutrophils (repair or damage) still needs to be explored. Moving forward, we should focus more on the functional significance of neutrophil-induced Slc7a11 in ALI/ARDS and its potential subsequent phenotypes.

In conclusion, we integrated two GEO datasets of ALI to comprehensively analyze the immune infiltration characteristics and mechanisms of ferroptosis-related genes. We identified three immune-mediated ferroptosis genes, namely, Cp, Slc7a11 and Slc39a14, which possibly regulated by neutrophils during the development of ALI, and their pathways may be involved in anti-oxidative stress and anti-lipid metabolism. Thus, the present study contributes to the understanding of ALI/ARDS and provide novel targets for future immunotherapeutic.

## Supplementary Information

**Additional file 1.** Full Western blot images.

**Additional file 2. **Methods. **Table S1.** The specific primer sequence list. **Table S2.** Antibodies used for immunofluorescence and western blot.

**Additional file 3: Figure S1.** Protein–protein networkbased on ferroptosis related genes in GSE2411 and GSE109913 datasets by Cytospace.GSE2411 dataset screened for 13 hub genes;GSE109913 dataset screened for 12 hub genes. **Figure S2.** Heatmap of CP, SLC7A11, SLC39A14 expression in GSE17355 dataset. **Figure S3.** Correlation analysis of SLC7A11 levels in BALF with different immune indicators.

**Additional file 4: Table S3.** The list of Ferroptosis-Related gene in acute lung injury. **Table S4.** DE-FRG list in GSE2411. **Table S5.** DE-FRG list in GSE109913. **Table S6.** Results of the correlation analysis between Cp and immune cells. **Table S7.** Results of the correlation analysis between Slc7a11 and immune cells. **Table S8.** Results of the correlation analysis between Slc39a14 and immune cells. **Table S9.** Pearson analysis with SLC7A11 levels in BALF.

## Data Availability

Not applicable, data was collected from public data repositories.

## Abbreviations

ALI: Acute lung injury
ARDS: Acute respiratory distress syndrome
GEO: Gene Expression Omnibus
FC: Fold-change
DEGs: Differentially expressed genes
FRG: Ferroptosis-related gene
qRT-PCR: Real-time quantitative PCR
CC: Cellular component
MF: Molecular function
BP: Biological process
GSEA: Gene Set Enrichment Analysis
LASSO: Least absolute shrinkage and selection operator regression
SVM: Support vector machine algorithm
GSEA: Gene set enrichment analysis
BALF: Bronchoalveolar lavage fluid
GO: Gene Ontology
KEGG: Kyoto Encyclopedia of Genes and Genomes
APACHE II: Acute Physiology and Chronic Health Evaluation II
BMI: Body mass index
SOFA: Sepsis-related organ failure assessment
ATCC: American Type Culture Collection
MLE-12: Mouse lung epithelial cell-12
MDA: Metabolite malondialdehyde
GSH: Glutathione
IF: Immunofluorescence
