# Supplementary material for: Ferroptosis-related signature and immune infiltration characterization in acute lung injury/acute respiratory distress syndrome
Source: Respir Res. 2023 Jun 10;24:154. doi: 10.1186/s12931-023-02429-y (PMC10257327; doi:10.1186/s12931-023-02429-y)

Full Western blot images

Figure 4G CP 128kd

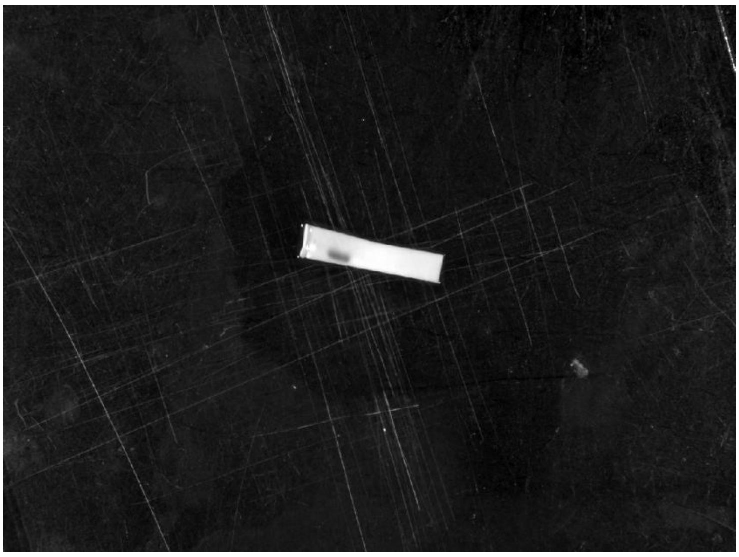

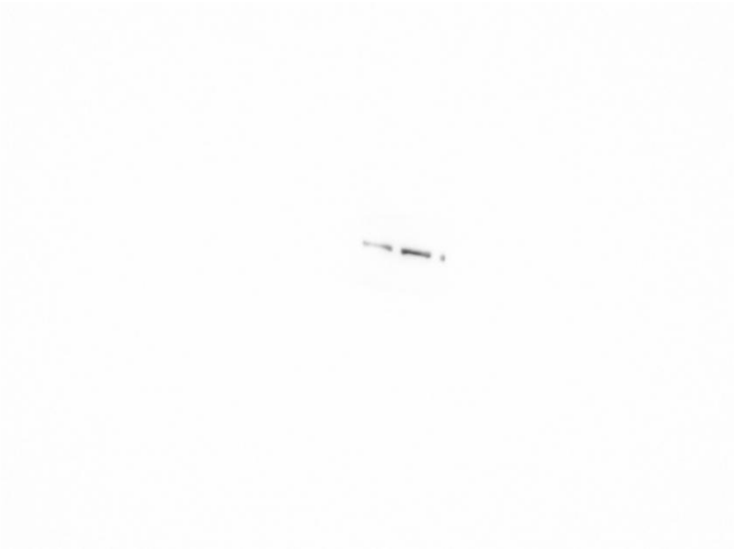

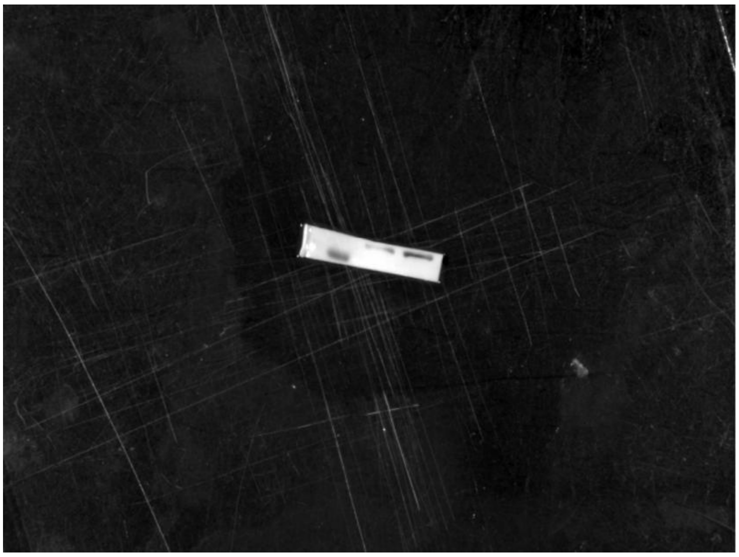

Figure 4G   Slc7a11 55kd

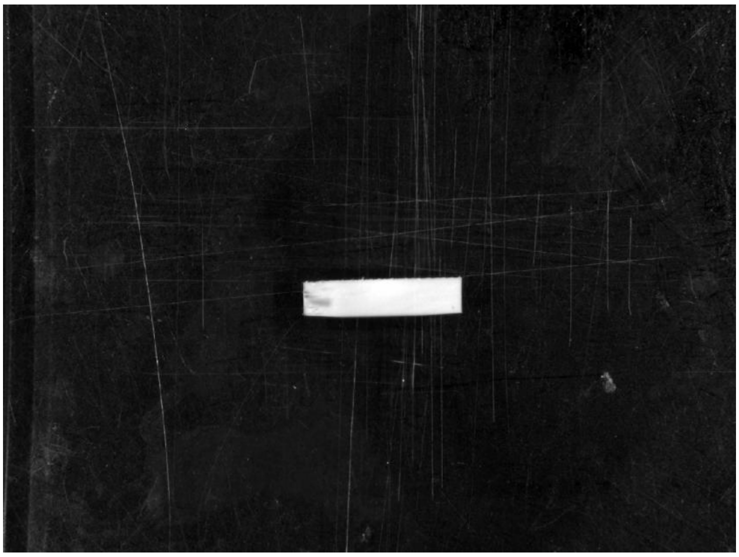

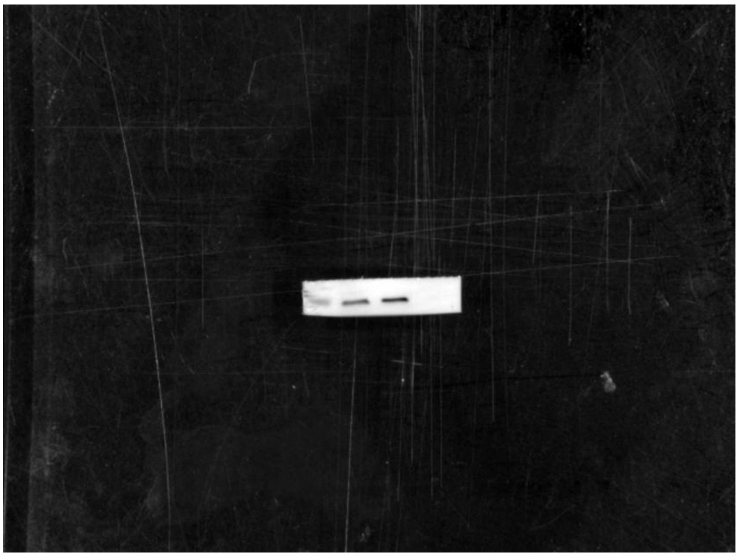

Figure 4G Slc39a14 57kd

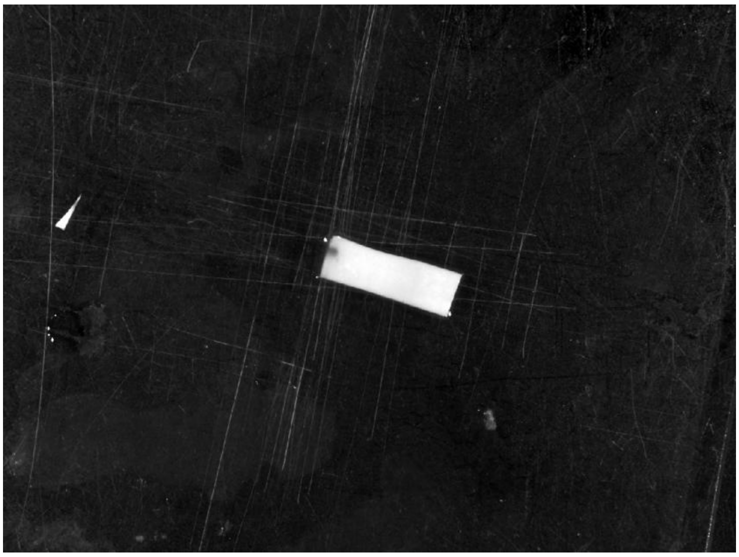

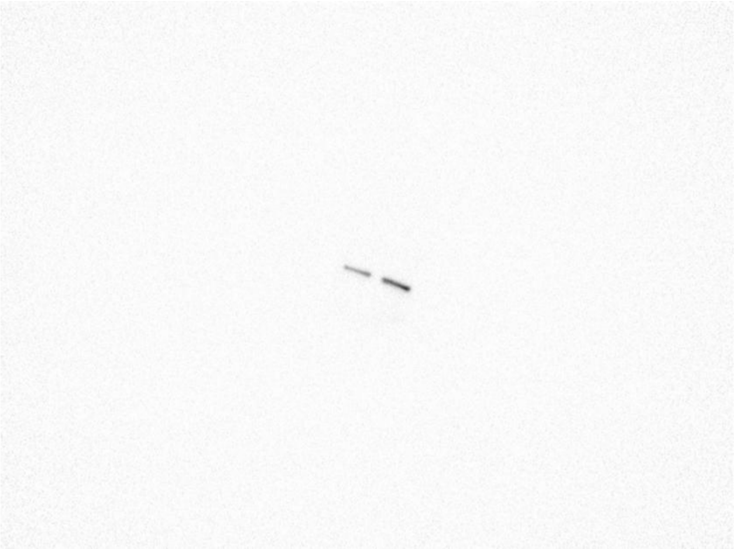

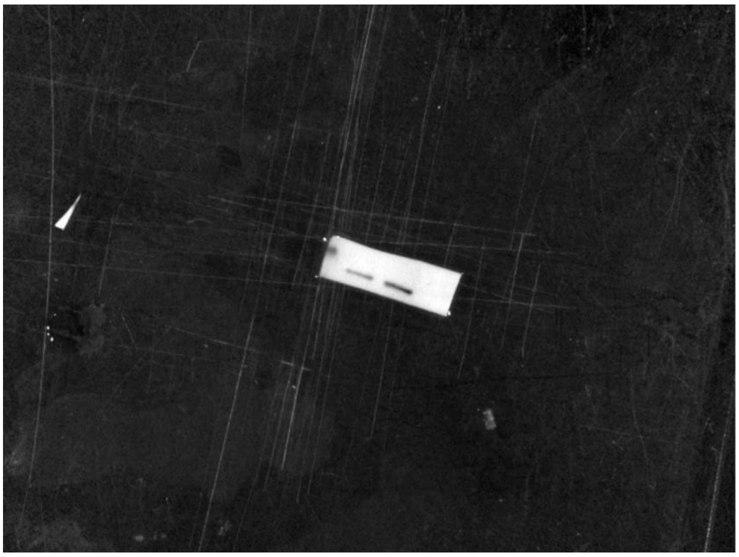

Figure 4G   Gpx4 22kd

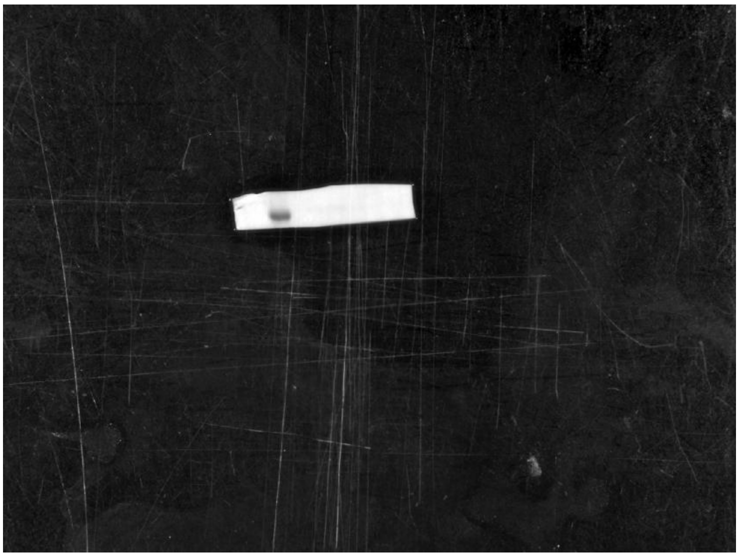

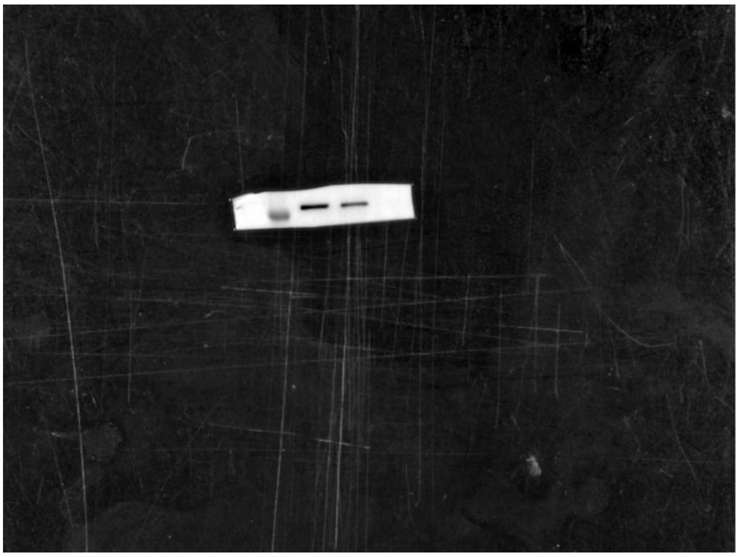

Figure 4G   Gapdh 37kd

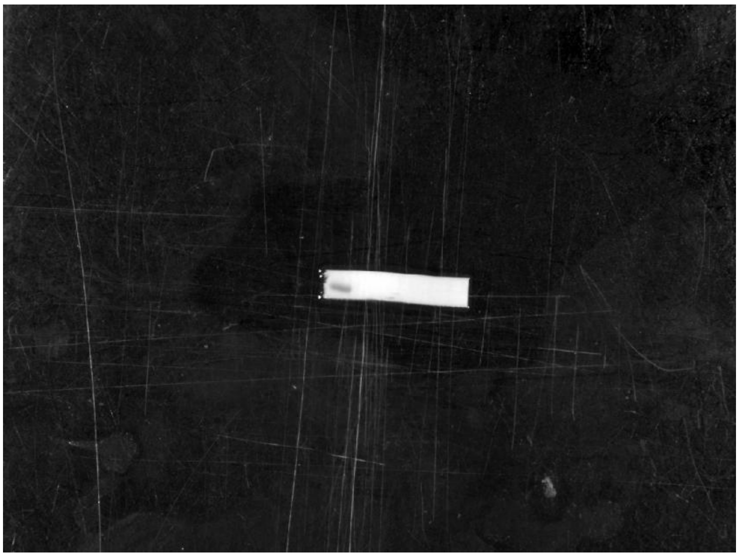

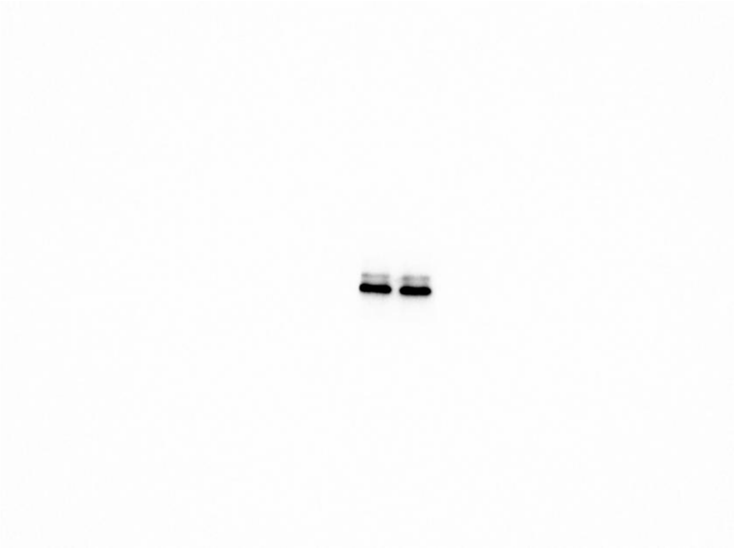

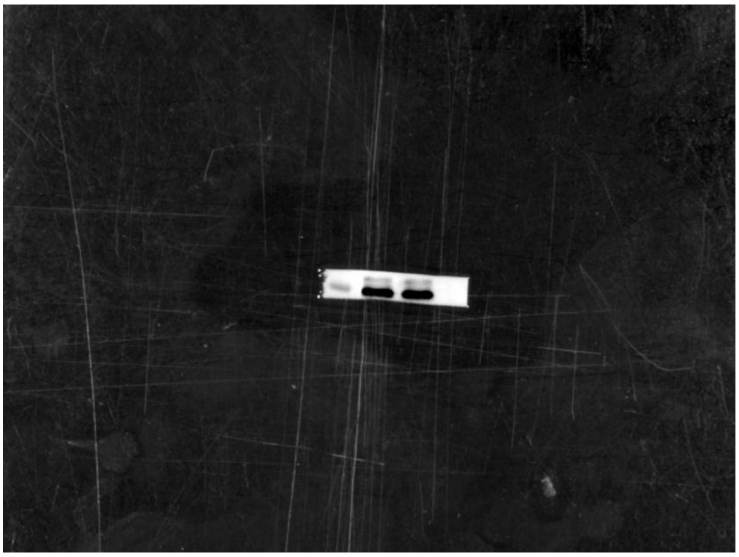

Figure 6E CP 128kd

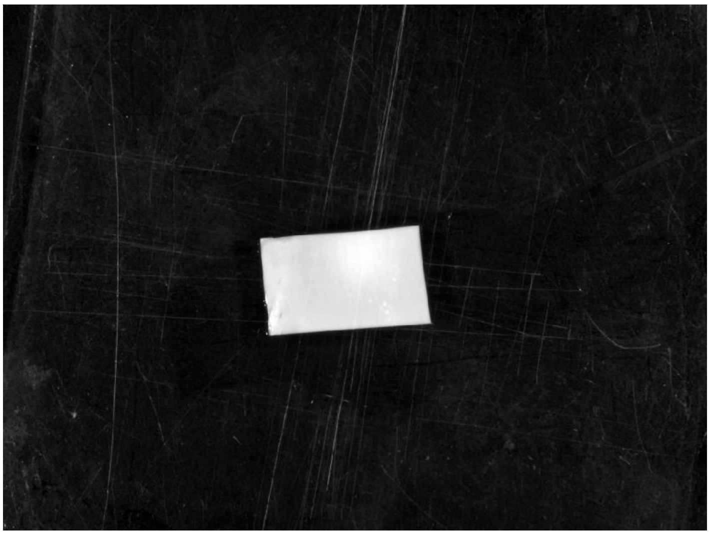

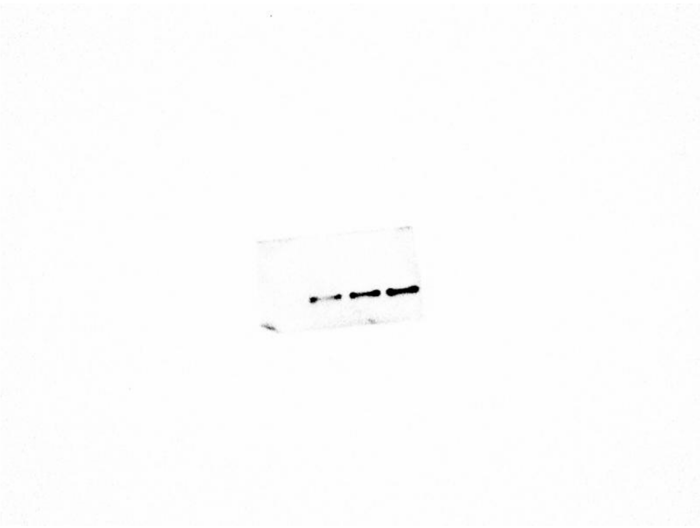

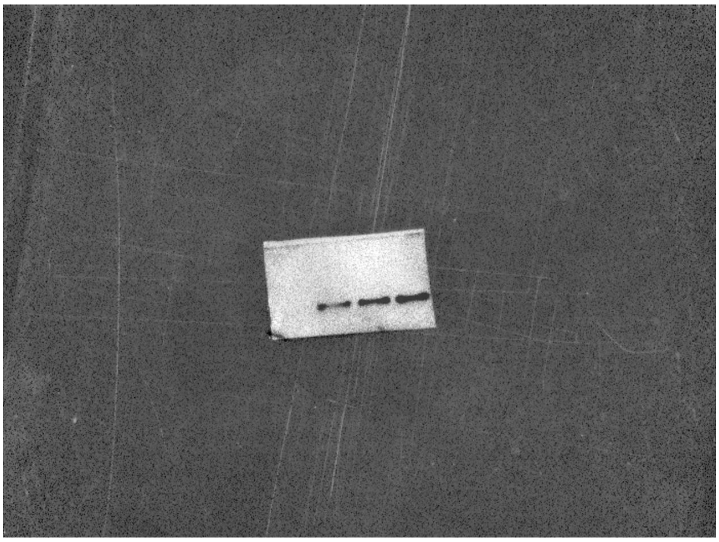

Figure 6E Slc7a11 55kd

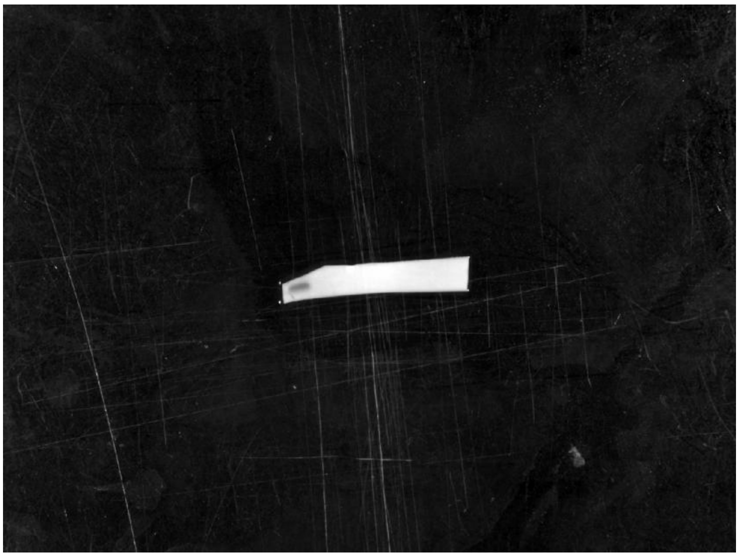

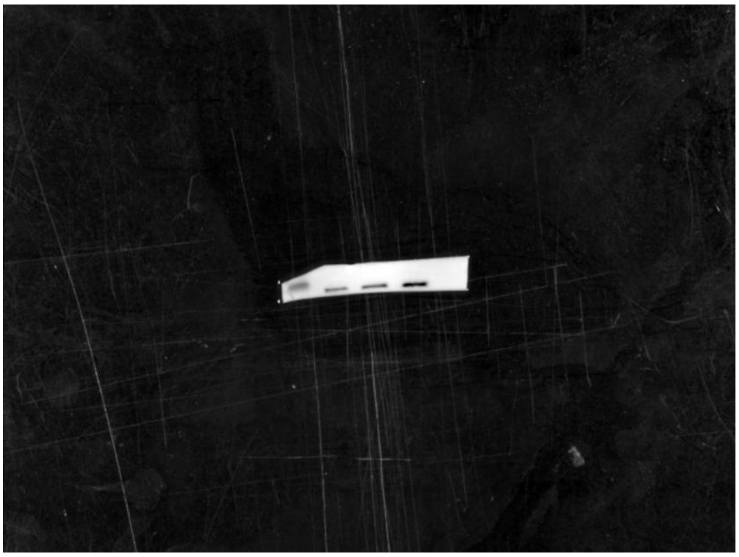

Figure 6E Slc39a14 57kd

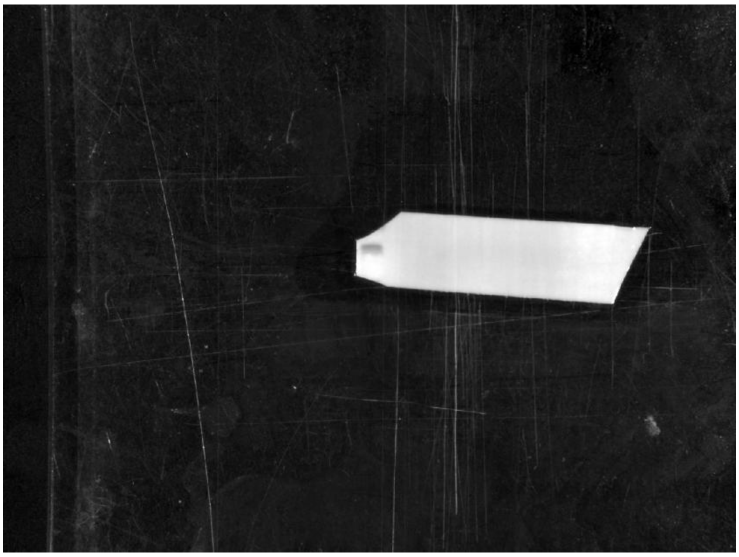

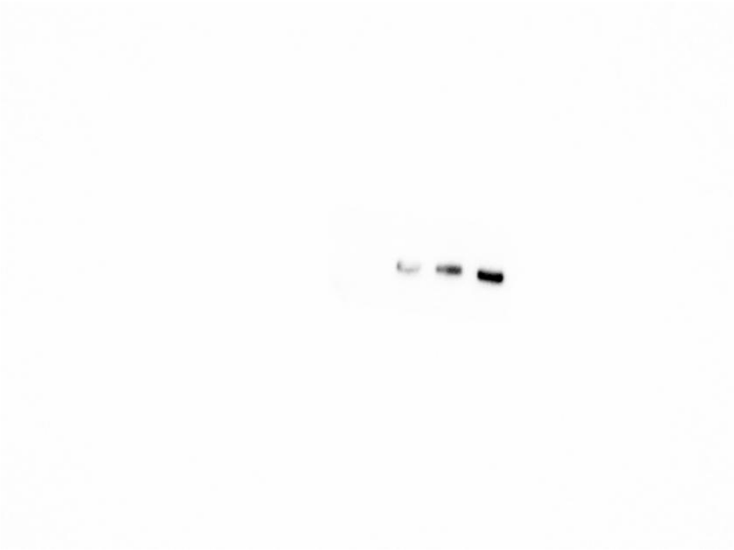

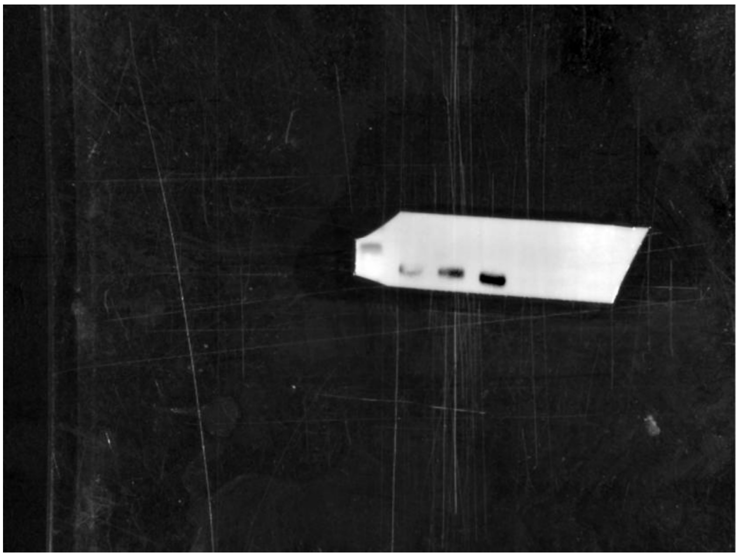

Figure 6E Gapdh 37kd

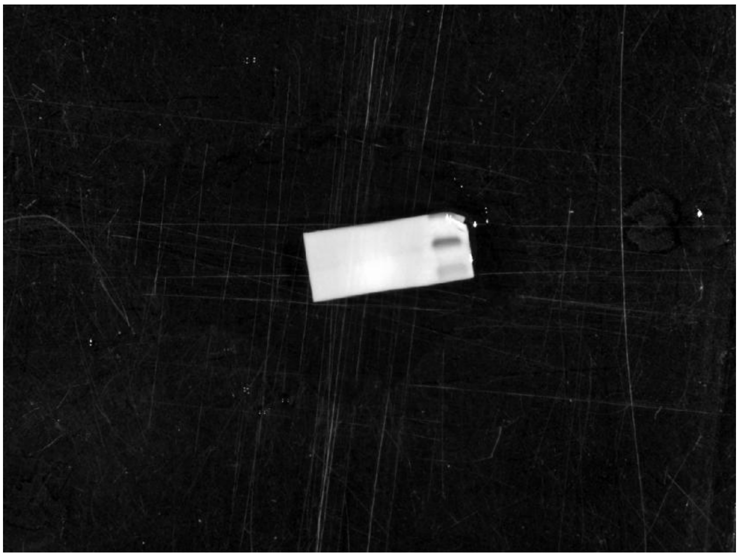

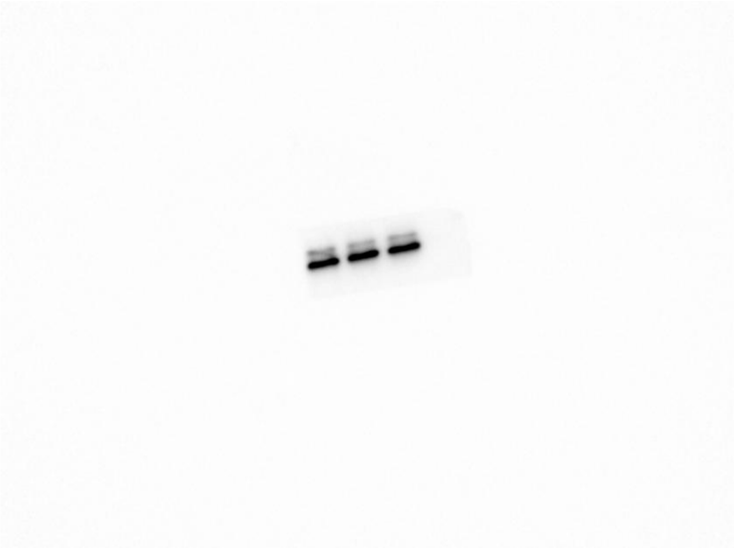

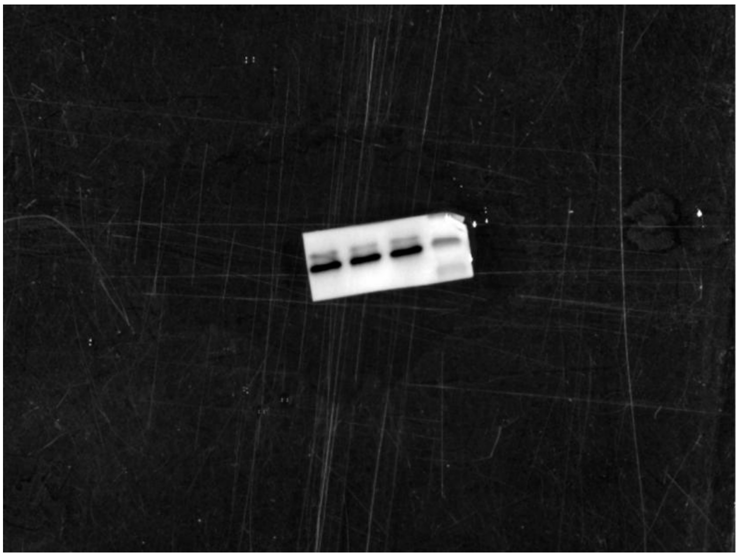

Figure7F CP 128kd

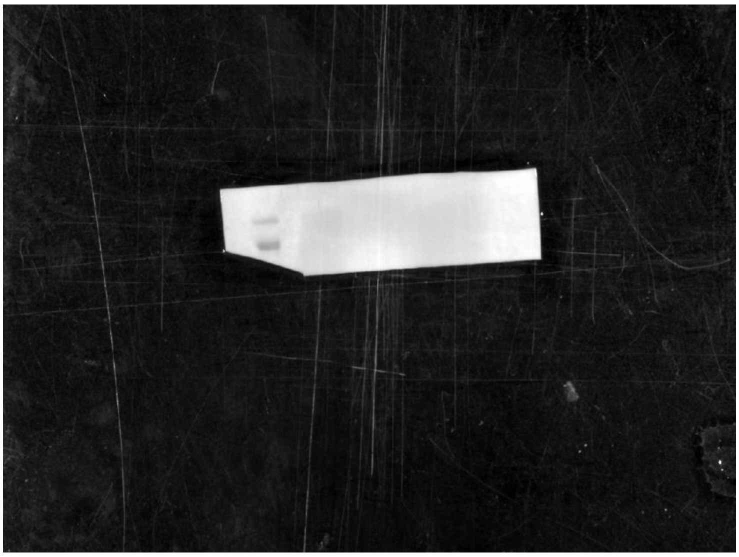

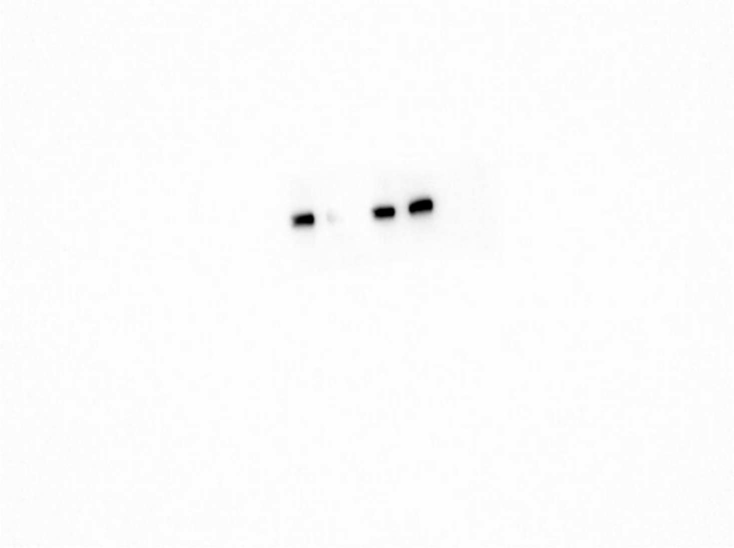

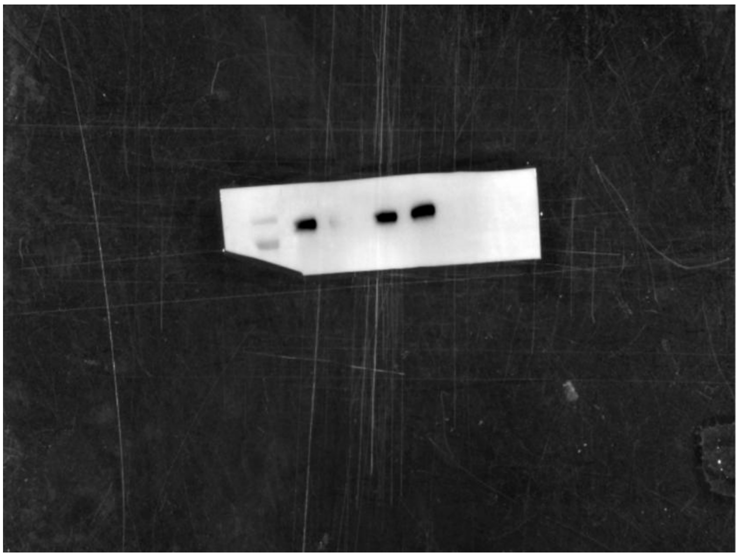

Figure7F Slc7a11 55kd

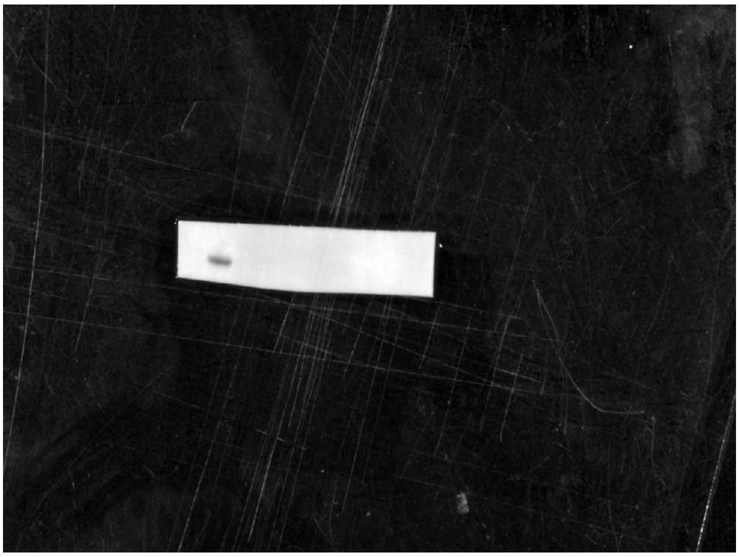

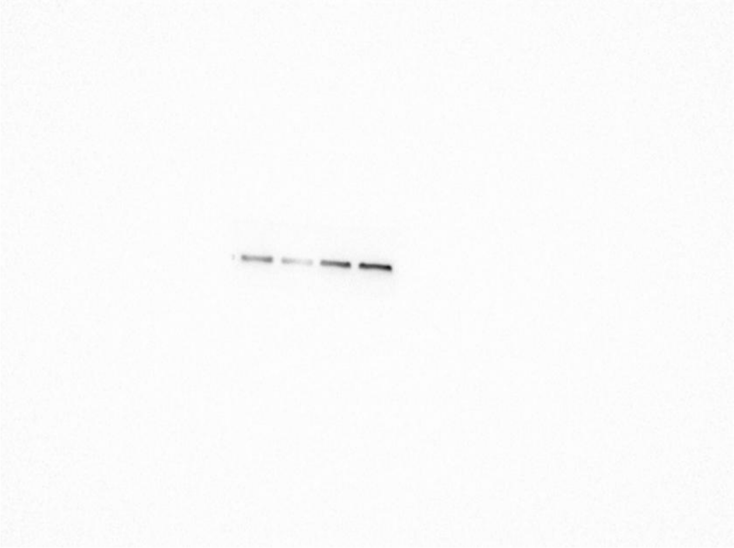

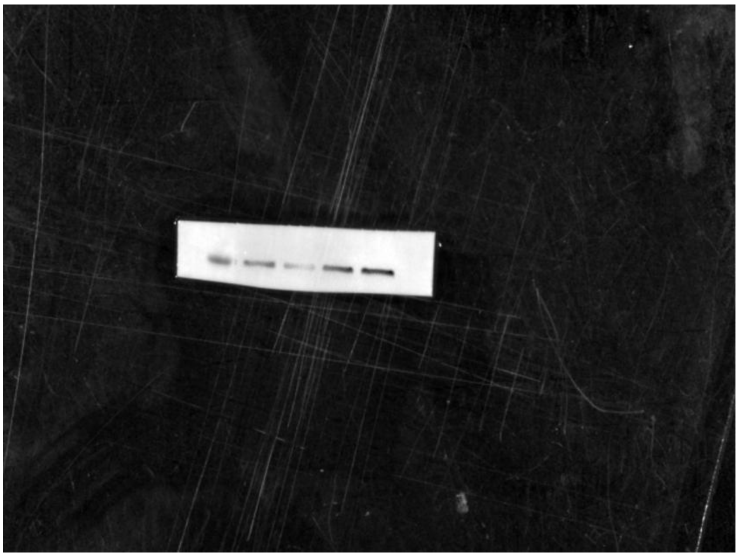

Figure7F Slc39a14 57kd

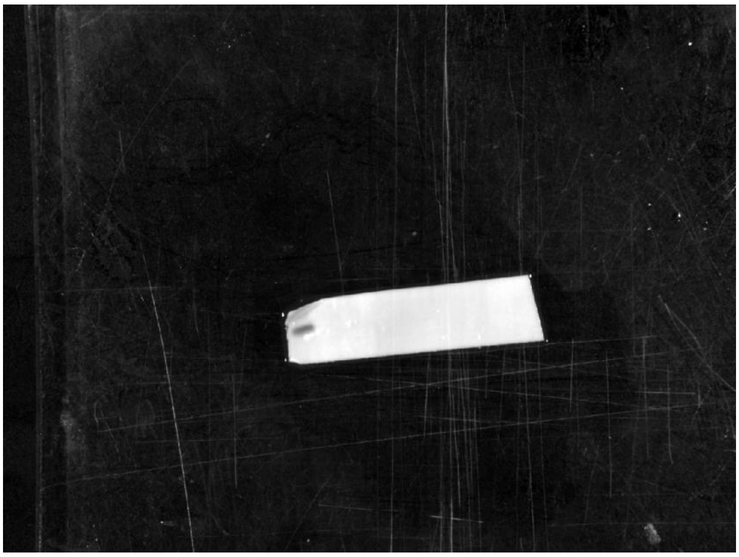

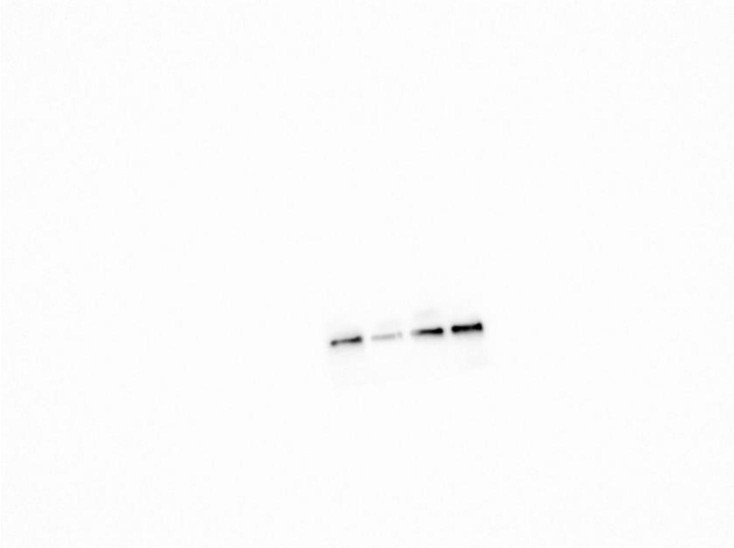

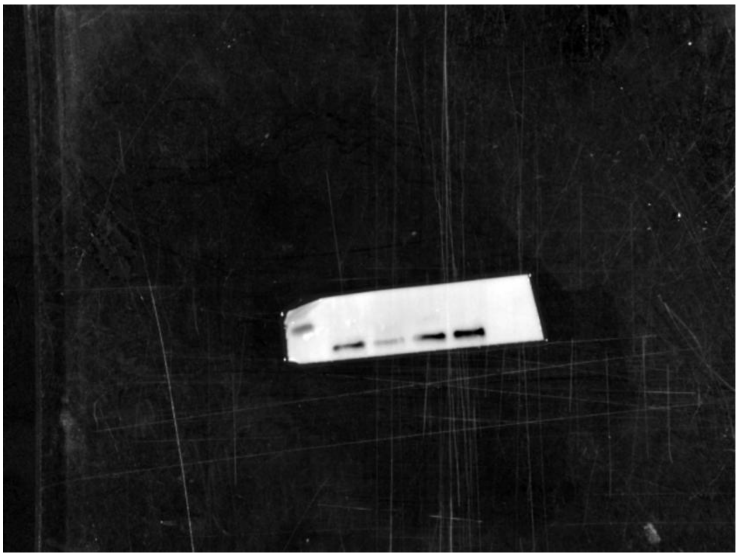

Figure7F Gpx4 22kd

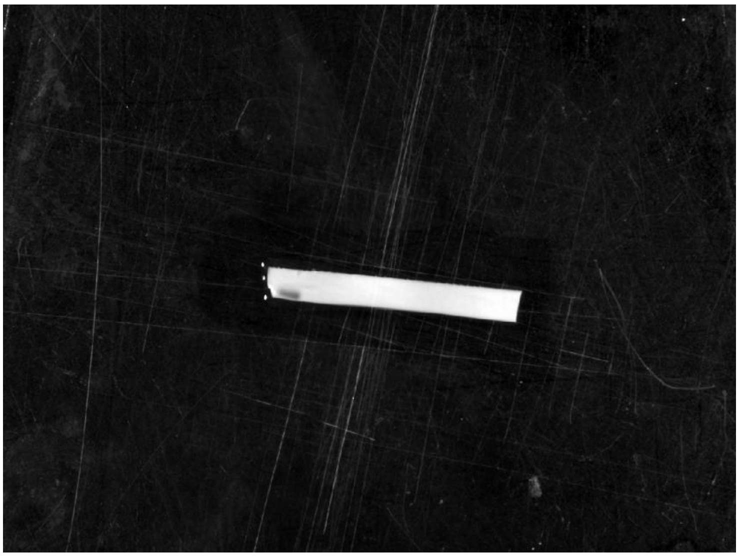

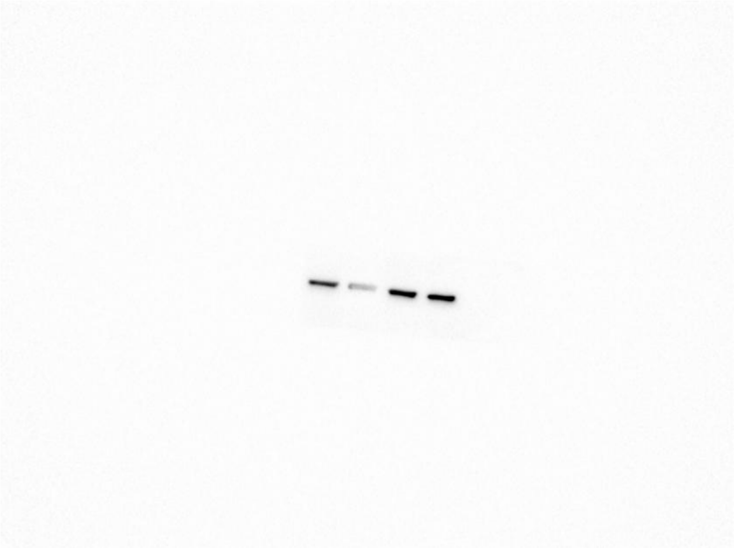

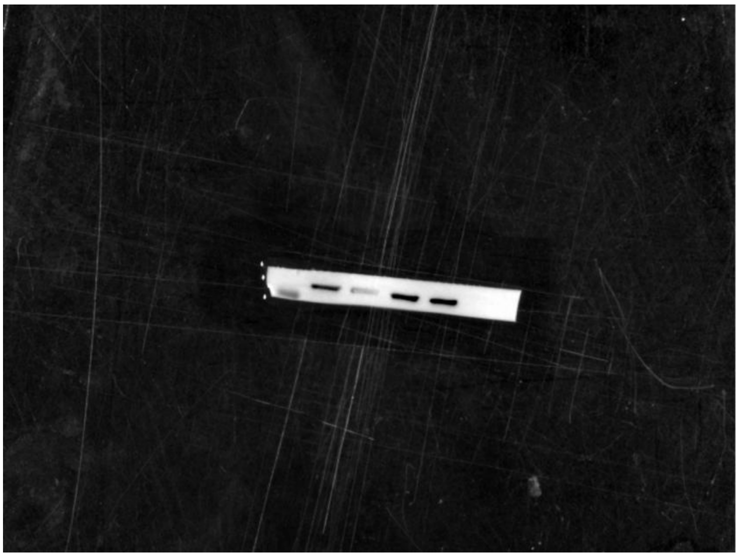

Figure7F   Gapdh 37kd

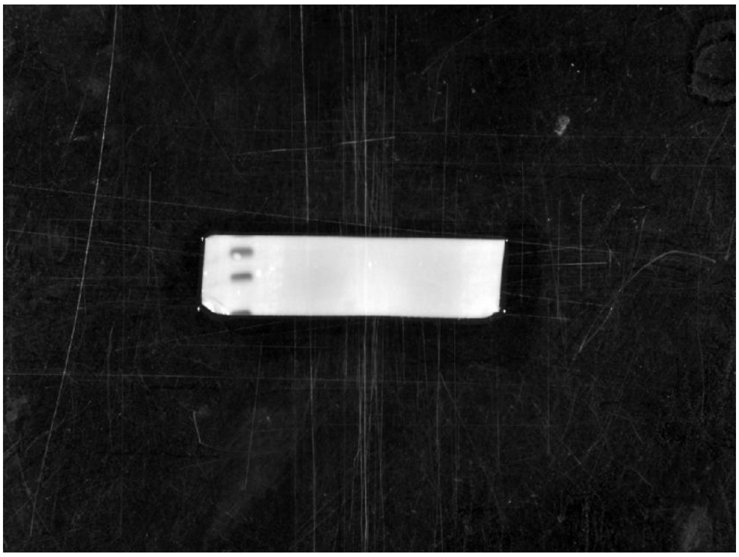

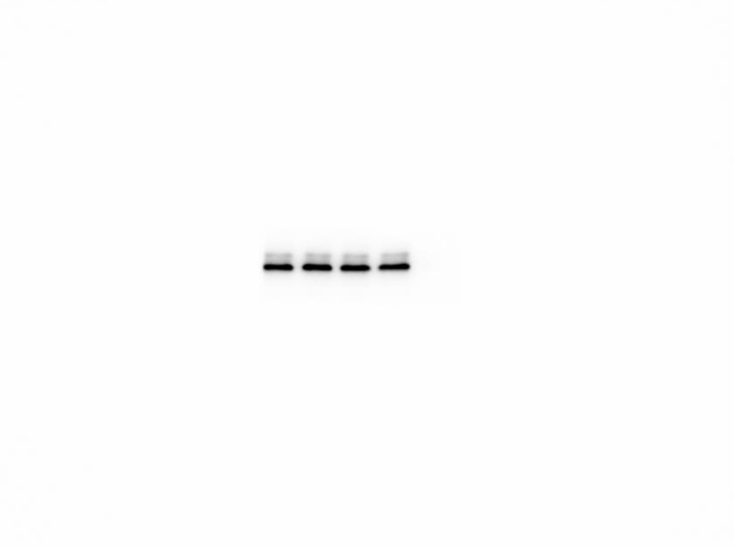

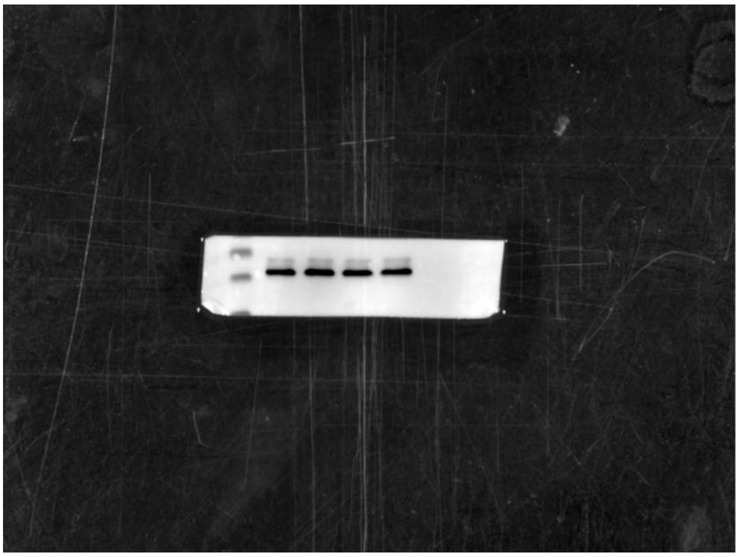

Supplement: Supplementary file 1 — Additional file 1. Full Western blot images. [file 12931_2023_2429_MOESM1_ESM.pdf]
