# Supplementary material for: Ferroptosis-related signature and immune infiltration characterization in acute lung injury/acute respiratory distress syndrome
Source: Respir Res. 2023 Jun 10;24:154. doi: 10.1186/s12931-023-02429-y (PMC10257327; doi:10.1186/s12931-023-02429-y)
Supplement: Supplementary file 2 — Additional file 2. Methods. Table S1. The specific primer sequence list. Table S2. Antibodies used for immunofluorescence and western blot. [file 12931_2023_2429_MOESM2_ESM.docx]

**Additional file 2**

**Methods**

***Elisa***

The expression of CP (MU30288, Bioswamp, Wuhan, China), SLC7A11(MU13303, Bioswamp, Wuhan, China), SLC39A14 (MU13310, Bioswamp, Wuhan, China) and GPX4 (MU31130, Bioswamp, Wuhan, China) in the patient's BALF was determined using the ELISA kits. The standards were diluted to different concentration gradients according to the instructions. Add 40μl of sample to the wells of the enzyme plate, followed by 10μl of biotin-labelled antibody to the key gene, and then add the sample to the bottom of the wells of the enzyme plate without touching the walls of the wells, shaking gently to mix. Add 50 μl of enzyme reagent to each well, except for the blank wells. Seal the plate with sealing film and incubate at 37°C for 30 minutes. After thorough washing, add 50μl of Chromogenic Agent A and then 50μl of Chromogenic Agent B to each well, shake gently and mix well, and develop for 10 minutes at 37°C, protected from light. Terminate the reaction by adding 50 μl of Terminating Solution to each well. The OD value of each well was measured sequentially at 450nm.

***Isolation of primary neutrophils***Neutrophils were isolated using mouse peripheral blood neutrophil isolation kit (TBD LZS1100). The anticoagulant mouse peripheral blood (800ul) and erythrocyte sedimentation solution were mixed in 1:1 ratio, and then carefully added to the upper layer of the separation solution with concentration gradient. Centrifuge 800g for 20min. At this time, blood should be divided into six layers: plasma, monocyte layer, separation fluid, neutrophil, other separation fluid, and erythrocyte sedimentation layer. Carefully suck the neutrophil layer and add the cleaning solution. Centrifuge 400g for 10min. Discard the supernatant after centrifugation, and remove red blood cells with red blood cell lysate. After centrifugation, the neutrophils were resuspended in 1640 medium for test.

***Flow cytometry***

The isolated neutrophils were lysed to remove red blood cells, centrifuged at 350xg for 5 minutes, and the supernatant was discarded. Non-specific immunofluorescence staining was reduced by blocking Fc receptor reagent. Add anti-Ly6g-FITC to stain the cell surface antibody, and incubate in the dark on the ice for 15-20 minutes. After centrifugation of 350g for 5 minutes, wash with at least 2ml of cell staining buffer. In 0.5ml of cell staining buffer, stir the cell particles again, and add 5 µ l/million cells of 7-AAD vitality staining solution to eliminate dead cells. After incubation on ice in the dark for 3-5 minutes, flow cytometry was performed by CytoFLEX Flow Cytometer.

***RNA isolation and quantitative real-time PCR (qRT‒PCR)***

Lung samples from mouse were rapidly frozen in liquid nitrogen and RNA extraction was performed with TRIzol. After reverse transcription of unstable RNA using the Takara RR036A Reverse Transcription Kit, qRT-PCR was performed using the Vazyme TB Green Premix Ex TaqTM II kit to further amplify mRNA for quantitative analysis of the expression of each gene. All expression data were normalized to 18S using the relative quantification method as an internal control.

**Table S1 The specific primer sequence list.**

| **Gene name** | **Sequence** |
| --- | --- |
| Cp | Forward CTTAGCCTTGGCAAGAGATAAGC |
|  | Reverse GGCCTAAAAACCCTAGCCAGG |
| Slc7a11 | Forward GGCACCGTCATCGGATCAG |
|  | Reverse CTCCACAGGCAGACCAGAAAA |
| Slc39a14 | Forward GTGTCTCACTGATTAACCTGGC |
|  | Reverse AGAGCAGCGTTCCAATGGAC |
| Gpx4 | Forward GCCTGGATAAGTACAGGGGTT |
|  | Forward CATGCAGATCGACTAGCTGAG |
| 18s | Forward GCAATTATTCCCCATGAACG |
|  | Reverse GGCCTCACTAAACCATCCAA |

***Immunofluorescence and Western blot***

The assays for immunofluorescence, western blot, and IHC were performed using the antibodies listed in Table S2. For the immunofluorescence assays, the SLC7A11, Ly6g and GPX4 were incubated with primary antibodies at 4°C overnight before being incubated with the corresponding secondary antibodies. Fluorescence images were acquired using a fluorescence microscope (Zeiss, Germany)

For western blot analysis, 20μg of fresh protein was extracted from lung tissue and cells and separated on SDS-PAGE gels, then transferred to a PVDF membrane which was incubated with the indicated primary antibodies. GAPDH was used as an internal reference.

**Table S2 Antibodies used for immunofluorescence(IF) and western blot (WB).**

| Antibodies | Manufacturer | Catalog number | IF  Dilusion | WB  Dilusion |
| --- | --- | --- | --- | --- |
| SLC7A11 | HUABIO | HA601071 | 1:200 | 1:1000 |
| SLC39A14 | ThermoFisher | PA5-21077 | / | 1:1000 |
| CP | Proteintech | 66156-1-Ig | / | 1:1000 |
| GPX4 | HUABIO | ET1706-45 | 1:200 | 1:1000 |
| GADPH | HUABIO | M1310-2 | / | 1:1000 |
| Ly6g | Biolegend | S19018G | 1:200 | / |

***Mitochondrial membrane potential***

Mitochondrial membrane potential was measured using JC-1 mitochondrial membrane potential assay dye (Elabscience, E-CK-A301), following the manufacturer's instructions. Briefly, groups of cells were washed using buffer and incubated with 10 μg/mL JC-1 solution for 10 min at 37°C in the dark, and results were obtained under microscopic (Carl Zeiss, Germany) observation.

***Transmission electron microscopy***

Lung tissues were minced into small pieces and washed with PBS and then fixed in 2.5% glutaraldehyde at 4°C overnight. The ultrastructural changes of ferroptosis in lung tissue were examined by transmission electron microscopy (Hitachi H-7800, Hitachi, Naka, Japan), which was performed by Hubei BIOSSCI Biotech Co., Ltd.
