# Supplementary material for: Ferroptosis-related signature and immune infiltration characterization in acute lung injury/acute respiratory distress syndrome
Source: Respir Res. 2023 Jun 10;24:154. doi: 10.1186/s12931-023-02429-y (PMC10257327; doi:10.1186/s12931-023-02429-y)

**Additional file 3**

**Figure S1 Protein-protein network (PPI) based on ferroptosis related genes in GSE2411 and GSE109913 datasets by Cytospace.** (A) GSE2411 dataset screened for 13 hub genes (yellow); (B) GSE109913 dataset screened for 12 hub genes (yellow).


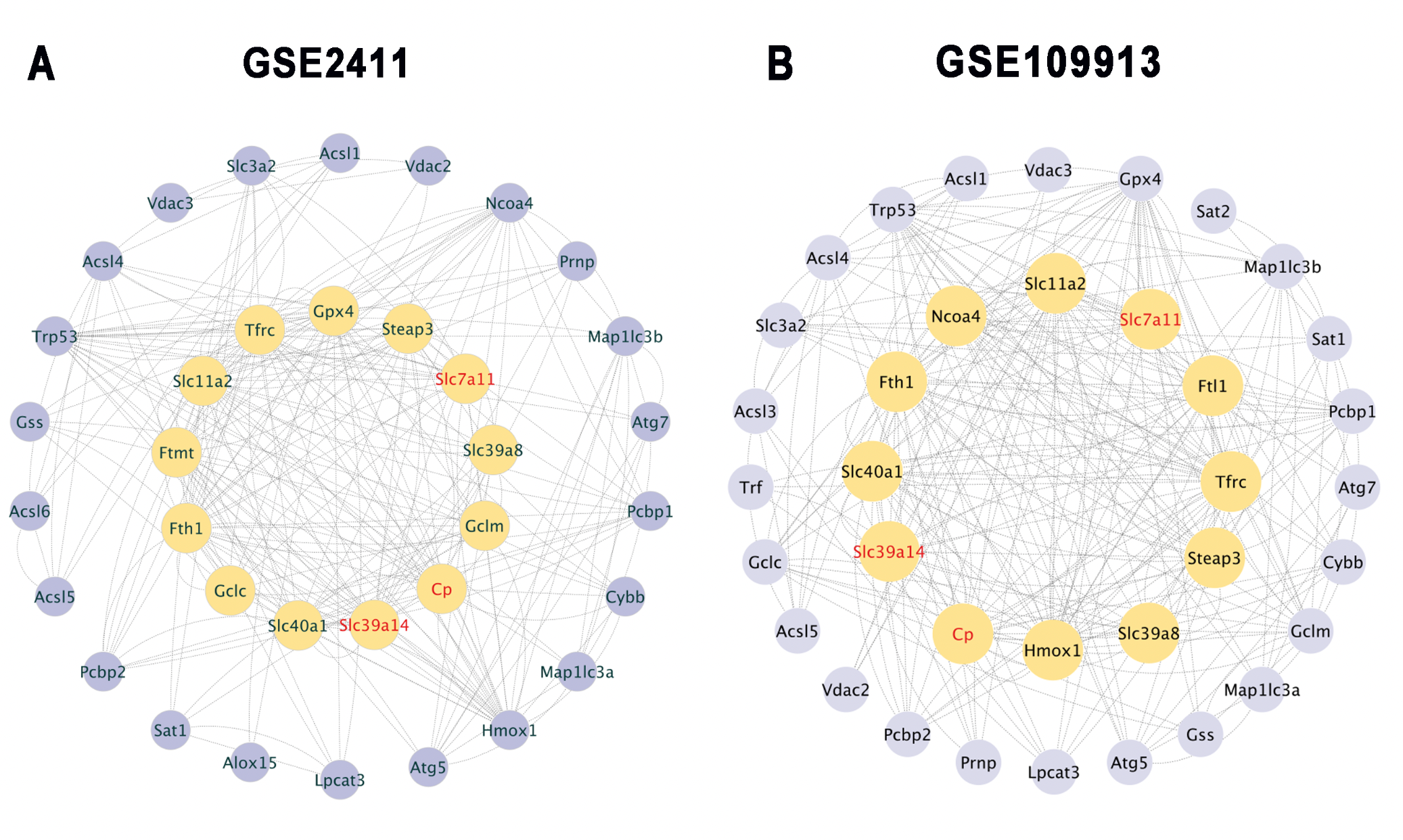


**Figure S2 Heatmap of CP, SLC7A11, SLC39A14 expression in GSE17355 dataset.**


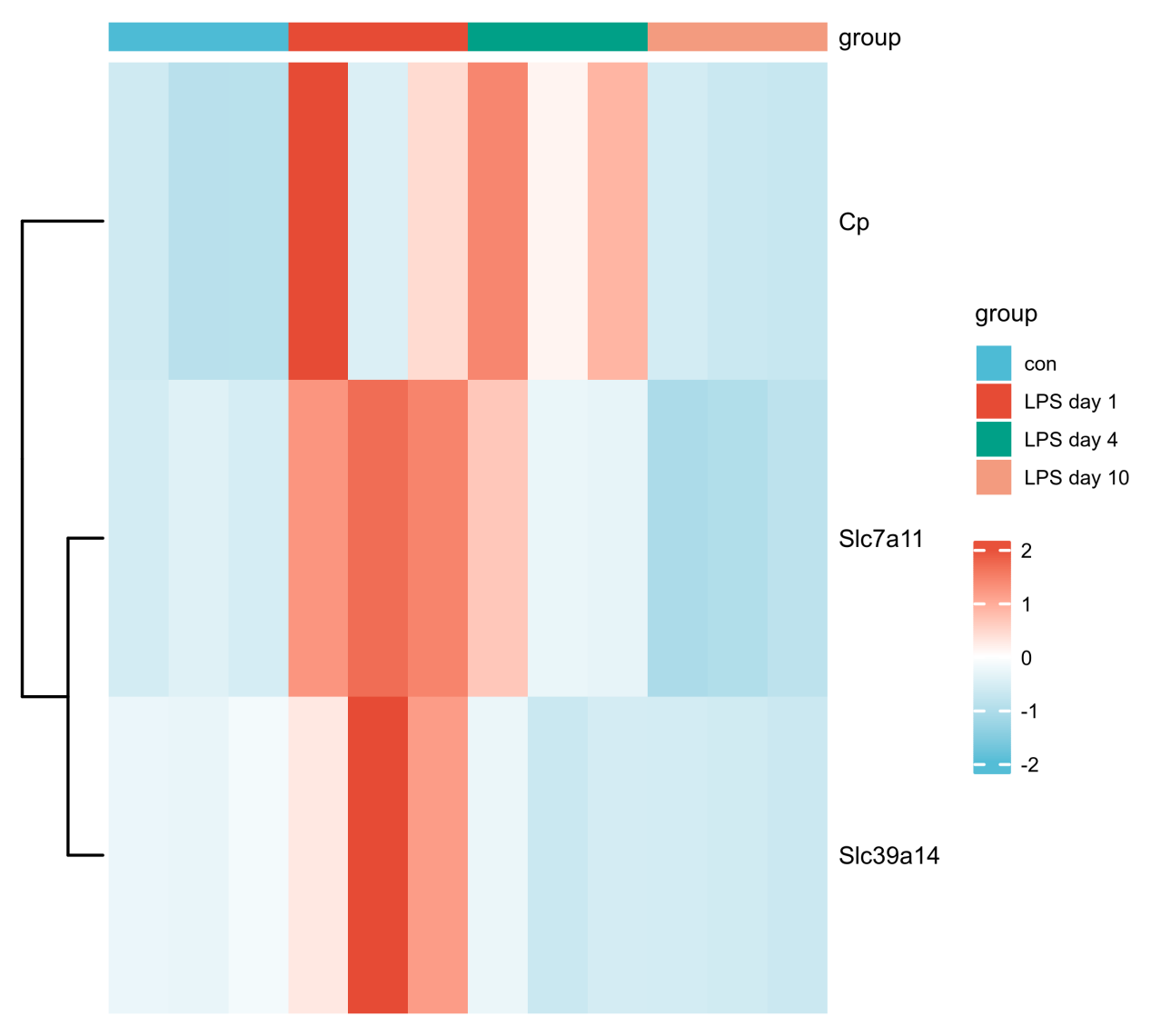


**Figure S3 Correlation analysis of SLC7A11 levels in BALF with different immune indicators.**


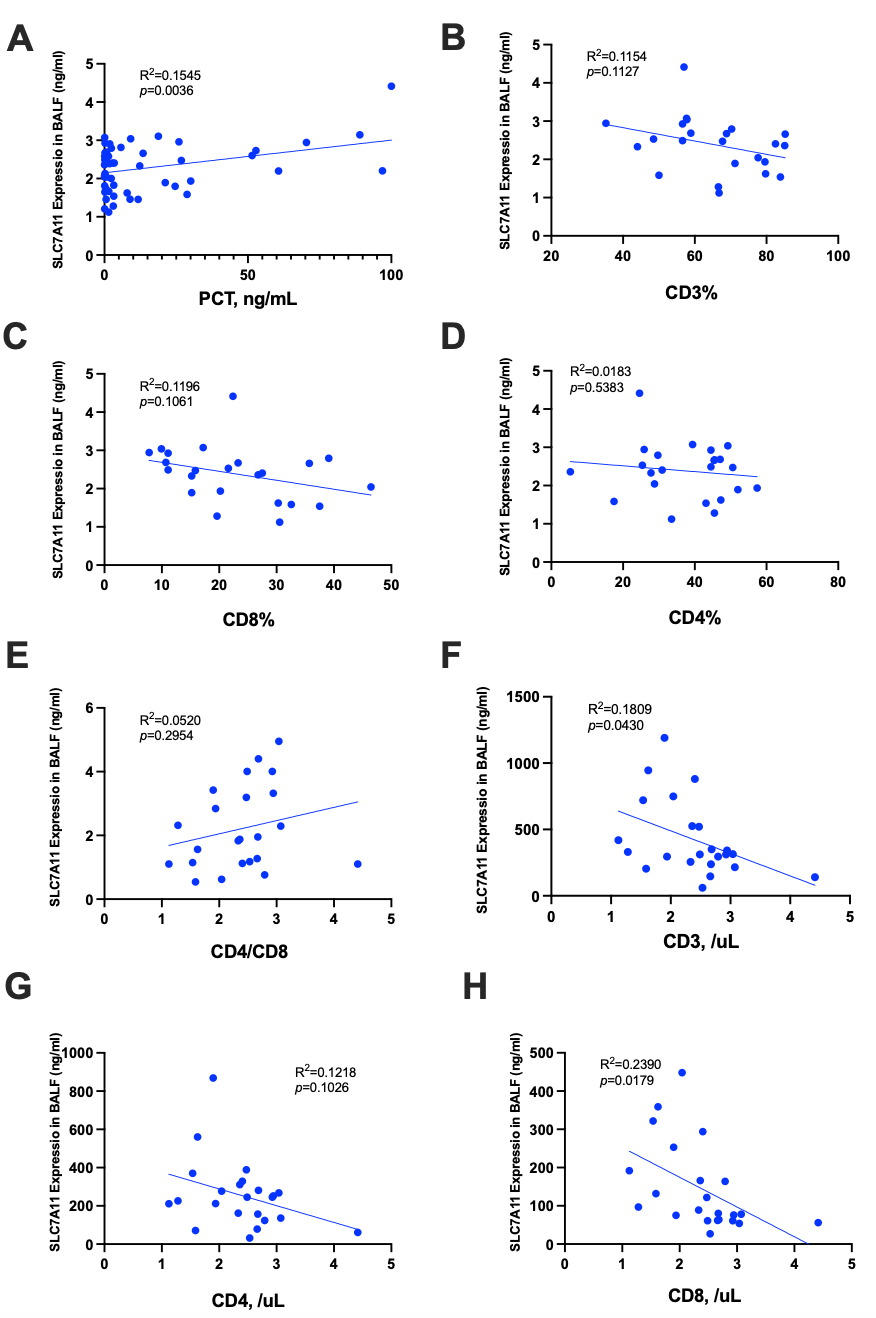

Supplement: Supplementary file 3 — Additional file 3: Figure S1. Protein–protein networkbased on ferroptosis related genes in GSE2411 and GSE109913 datasets by Cytospace.GSE2411 dataset screened for 13 hub genes;GSE109913 dataset screened for 12 hub genes. Figure S2. Heatmap of CP, SLC7A11, SLC39A14 expression in GSE17355 dataset. Figure S3. Correlation analysis of SLC7A11 levels in BALF with different immune indicators. [file 12931_2023_2429_MOESM3_ESM.docx]
