# Supplementary material for: Ferroptosis-related signature and immune infiltration characterization in acute lung injury/acute respiratory distress syndrome
Source: Respir Res. 2023 Jun 10;24:154. doi: 10.1186/s12931-023-02429-y (PMC10257327; doi:10.1186/s12931-023-02429-y)
Supplement: Supplementary file 4 — Additional file 4: Table S3. The list of Ferroptosis-Related gene in acute lung injury. Table S4. DE-FRG list in GSE2411. Table S5. DE-FRG list in GSE109913. Table S6. Results of the correlation analysis between Cp and immune cells. Table S7. Results of the correlation analysis between Slc7a11 and immune cells. Table S8. Results of the correlation analysis between Slc39a14 and immune cells. Table S9. Pearson analysis with SLC7A11 levels in BALF. [file 12931_2023_2429_MOESM4_ESM.docx]

| **Table S3 The list of Ferroptosis-Related gene in acute lung injury.** | | | | |
| --- | --- | --- | --- | --- |
| Slc3a2 | Trf | Atg5 | Acsl6 | Cp |
| Slc7a11 | Inhca | Atg7 | Acsl3 | Pcbp1 |
| Trp53 | Tfrc | Ncoa4 | Acsl5 | Fth1 |
| Gclc | Steap3 | Prnp | Lpcat3 | Ftl1 |
| Gclm | Slc11a2 | Hmox1 | Alox15 | Map1lc3a |
| Gss | Slc39a8 | Vdac2 | Sat1 | Map1lc3b |
| Gpx4 | Slc39a14 | Vdac3 | Sat2 | Slc40a1 |
| Acsl1 | Pcbp2 | Cybb | Acsl4 | Ftmt |

| **Table S4 DE-FRG list in GSE2411.** | | | | | | |
| --- | --- | --- | --- | --- | --- | --- |
|  | logFC | AveExpr | t | P.Value | adj.P.Val | B |
| Acsl1 | -0.27056816 | 8.34686233 | -2.297173346 | 0.037997012 | 0.111319638 | -4.644260245 |
| Acsl4 | 0.047250844 | 8.56212804 | 0.527384822 | 0.606397535 | 0.764695258 | -6.86942909 |
| Acsl5 | -0.134388746 | 10.5060069 | -2.180606602 | 0.047249222 | 0.13156312 | -4.846875204 |
| Acsl6 | -0.020520397 | 5.197451765 | -0.479147134 | 0.639423495 | 0.79029666 | -6.89480339 |
| Alox15 | 0.01595393 | 7.22775621 | 0.17360737 | 0.864723707 | 0.93105391 | -6.999982315 |
| Atg5 | -0.187957209 | 6.960049404 | -3.079230535 | 0.008374366 | 0.035082855 | -3.192744033 |
| Atg7 | -0.025280441 | 8.449063189 | -0.599396934 | 0.558732118 | 0.728747824 | -6.827221758 |
| Cp | 0.44134721 | 10.1927313 | 5.23438214 | 0.000137584 | 0.00163285 | 0.932306951 |
| Cybb | 0.955789655 | 8.334804923 | 3.798383415 | 0.002042662 | 0.012049953 | -1.794480102 |
| Fth1 | 0.210315163 | 13.48094128 | 4.451596703 | 0.000582179 | 0.004667094 | -0.531356386 |
| Ftmt | -0.007912107 | 5.237917895 | -0.189336407 | 0.85261717 | 0.924259121 | -6.996950276 |
| Gclc | 1.233543339 | 11.68784605 | 7.744091583 | 2.35E-06 | 9.44E-05 | 5.075095016 |
| Gclm | 0.396711022 | 9.818949834 | 4.77587692 | 0.000317337 | 0.0029713 | 0.08321876 |
| Gpx4 | -0.061266274 | 11.10972312 | -0.797983843 | 0.438550502 | 0.630911372 | -6.68471585 |
| Gss | -0.126492352 | 7.902047612 | -3.155318119 | 0.00721125 | 0.031310365 | -3.046074339 |
| Hmox1 | 0.515831269 | 8.510069344 | 3.250300882 | 0.005982574 | 0.027197879 | -2.862272089 |
| Lpcat3 | -0.216633475 | 9.523400032 | -3.818810564 | 0.001962987 | 0.01168031 | -1.75466344 |
| Map1lc3a | -0.122508202 | 10.30527251 | -1.098742394 | 0.29089377 | 0.488996963 | -6.400338451 |
| Map1lc3b | -0.171579679 | 10.70179962 | -3.788148141 | 0.002083814 | 0.012253916 | -1.814434388 |
| Ncoa4 | 0.031429541 | 9.822185035 | 0.253709323 | 0.803503804 | 0.895779709 | -6.981821714 |
| Pcbp1 | 0.090504598 | 10.91688554 | 1.569816747 | 0.13935511 | 0.295066346 | -5.810725818 |
| Pcbp2 | -0.131100321 | 10.635815 | -2.555807793 | 0.023221537 | 0.07691176 | -4.179291359 |
| Prnp | -0.002120686 | 10.78207975 | -0.032568906 | 0.974490073 | 0.988173728 | -7.015446491 |
| Sat1 | 0.547103394 | 10.56196636 | 4.971397526 | 0.000221464 | 0.002278044 | 0.448355871 |
| Slc11a2 | 0.321650905 | 7.997679599 | 3.641884466 | 0.00277292 | 0.015183542 | -2.099758381 |
| Slc39a14 | 1.28116665 | 9.006369333 | 11.80826403 | 1.52E-08 | 3.81E-06 | 10.11670341 |
| Slc39a8 | 0.311309092 | 10.41109154 | 2.602673128 | 0.021216627 | 0.071487592 | -4.093102635 |
| Slc3a2 | 0.293960565 | 11.66842 | 4.732180997 | 0.000344124 | 0.003151974 | 0.001032535 |
| Slc40a1 | -0.017539266 | 8.196016623 | -0.152865069 | 0.880742622 | 0.94044421 | -7.003580608 |
| Slc7a11 | 0.842877141 | 7.217995575 | 6.982327415 | 7.39E-06 | 0.000206523 | 3.910719492 |
| Steap3 | -0.043774058 | 6.726023663 | -1.045003262 | 0.314185596 | 0.513975006 | -6.456869908 |
| Tfrc | -0.212067845 | 7.538403239 | -2.747663627 | 0.016020056 | 0.057686791 | -3.823351385 |
| Trp53 | 0.328445645 | 7.425859071 | 2.66652204 | 0.018752792 | 0.065207322 | -3.974862673 |
| Vdac2 | -0.015878857 | 11.33034855 | -0.227029601 | 0.823767538 | 0.908386879 | -6.988621349 |
| Vdac3 | -0.017484273 | 10.13214059 | -0.193527102 | 0.849397994 | 0.922686602 | -6.996098342 |

| **Table S5 DE-FRG list in GSE109913.** | | | | | | |
| --- | --- | --- | --- | --- | --- | --- |
|  | logFC | AveExpr | t | P.Value | adj.P.Val | B |
| Acsl1 | 0.756752923 | 1.8908318 | 2.656693773 | 0.027369783 | 0.064132811 | -4.405796783 |
| Acsl3 | -1.489188839 | 2.939546438 | -5.946020003 | 0.000266209 | 0.002952643 | 0.453820829 |
| Acsl4 | -0.703725202 | 4.963170312 | -2.402993682 | 0.041106756 | 0.086688361 | -4.811979119 |
| Acsl5 | 0.049362992 | 6.194309572 | 0.305756529 | 0.767111482 | 0.822183083 | -7.208416457 |
| Atg5 | 0.030790526 | 3.725979446 | 0.202873646 | 0.843984585 | 0.882562772 | -7.237274851 |
| Atg7 | 0.296947496 | 2.204586064 | 1.517771062 | 0.165183818 | 0.246635476 | -6.127170325 |
| Cp | 1.169198082 | 5.828870269 | 5.749312075 | 0.000336775 | 0.003427009 | 0.203143228 |
| Cybb | 2.207066102 | 5.122597388 | 7.773023315 | 3.74E-05 | 0.000868226 | 2.542472966 |
| Fth1 | 1.821700833 | 12.0149808 | 5.714035195 | 0.00035147 | 0.003533995 | 0.157616197 |
| Ftl1 | 0.388847525 | 9.323518286 | 1.470538377 | 0.177274532 | 0.260636448 | -6.189285014 |
| Gclc | -1.118471117 | 5.42280589 | -3.22571244 | 0.011130028 | 0.034208333 | -3.487491769 |
| Gclm | -1.227983712 | 2.423200993 | -3.646128335 | 0.00584429 | 0.022397134 | -2.818265193 |
| Gpx4 | 0.149102657 | 7.490828763 | 0.756931993 | 0.46945763 | 0.562701854 | -6.951906328 |
| Gss | -0.326590321 | 2.264009781 | -1.589638414 | 0.148193381 | 0.226764269 | -6.030535151 |
| Hmox1 | 1.366210753 | 6.237763192 | 9.226486284 | 9.98E-06 | 0.000383774 | 3.938972635 |
| Lpcat3 | 0.175846404 | 3.392301714 | 0.964286167 | 0.361411645 | 0.457805861 | -6.769623429 |
| Map1lc3a | -0.549021522 | 6.858753581 | -3.068768941 | 0.014227451 | 0.040517112 | -3.74027749 |
| Map1lc3b | -0.241462109 | 4.245655535 | -1.663405866 | 0.132409308 | 0.208080538 | -5.92885946 |
| Ncoa4 | 0.165660565 | 3.622173491 | 0.646571235 | 0.534897629 | 0.623933667 | -7.033259476 |
| Pcbp1 | 0.257046892 | 6.81826353 | 1.360323372 | 0.208546843 | 0.295736813 | -6.329565846 |
| Pcbp2 | -0.176040697 | 6.338127611 | -0.968776854 | 0.35929076 | 0.455749777 | -6.765276363 |
| Prnp | -1.158933214 | 6.154968086 | -5.225912145 | 0.000645807 | 0.005233135 | -0.490493616 |
| Sat1 | -0.435746522 | 6.579360358 | -2.669394304 | 0.026819332 | 0.063194978 | -4.385337823 |
| Sat2 | -0.230227184 | 1.819340789 | -1.097852739 | 0.302256621 | 0.39763825 | -6.633610952 |
| Slc11a2 | 0.584830846 | 2.971175818 | 3.487064989 | 0.007438567 | 0.026136792 | -3.069763256 |
| Slc39a14 | 1.851251617 | 3.140569139 | 6.471029036 | 0.000145611 | 0.002002091 | 1.097049236 |
| Slc39a8 | -0.159421311 | 4.225774135 | -0.408439265 | 0.693001998 | 0.759890024 | -7.168268027 |
| Slc3a2 | 0.231493328 | 6.317198759 | 1.12099154 | 0.29282289 | 0.388253924 | -6.608685793 |
| Slc40a1 | 0.182013561 | 0.867465187 | 1.153698326 | 0.279889056 | 0.374289469 | -6.57279935 |
| Slc7a11 | 2.630462542 | 2.072943476 | 13.54646065 | 4.56E-07 | 6.26E-05 | 7.11628692 |
| Steap3 | -1.222271383 | 2.609752175 | -6.190295472 | 0.00020019 | 0.002468229 | 0.757704224 |
| Tfrc | -0.825005743 | 3.815230927 | -3.341897036 | 0.009295584 | 0.030233209 | -3.301192793 |
| Trf | -0.494564073 | 6.516849896 | -2.899838405 | 0.018577607 | 0.048798778 | -4.01320279 |
| Trp53 | 0.45254627 | 3.641920585 | 3.107723673 | 0.013383289 | 0.038895361 | -3.67743848 |
| Vdac2 | 0.52673875 | 4.660510882 | 3.458336143 | 0.007772427 | 0.026815946 | -3.115429918 |

| **Table S6 Results of the correlation analysis between Cp and immune cells.** | | |
| --- | --- | --- |
| cell | cor | p.value |
| Mast Cells | 0.06993007 | 0.829023641 |
| Neutrophil Cells | 0.706293706 | 0.010245194 |
| Eosinophil Cells | -0.836042088 | 0.000703706 |
| B Cells Memory | 0.094571073 | 0.770019583 |
| B Cells Naive | -0.325744808 | 0.301482697 |
| Plasma Cells | -0.321678322 | 0.307909876 |
| T Cells CD8 Actived | 0.172018152 | 0.59293728 |
| T Cells CD8 Naive | 0.220294765 | 0.491450645 |
| T Cells CD8 Memory | -0.202747921 | 0.527404884 |
| M0 Macrophage | -0.790487305 | 0.002209579 |
| M1 Macrophage | 0.454545455 | 0.137657916 |
| M2 Macrophage | 0.533892354 | 0.073792459 |
| Treg Cells | -0.545454545 | 0.066611882 |
| T Cells CD4 Memory | -0.342657343 | 0.275567451 |
| T Cells CD4 Naive | 0.486865895 | 0.108442822 |
| T Cells CD4 Follicular | NA | NA |
| Th1 Cells | 0.084063176 | 0.795062624 |
| Th17 Cells | -0.797202797 | 0.001900368 |
| Th2 Cells | NA | NA |
| Monocyte | -0.202797203 | 0.527302354 |
| GammaDelta T Cells | -0.692307692 | 0.01259303 |
| NK Resting | 0.123166915 | 0.70294168 |

| **Table S7 Results of the correlation analysis between Slc7a11 and immune cells.** | | |
| --- | --- | --- |
| cell | cor | p.value |
| Mast Cells | 0.006993007 | 0.982791769 |
| Neutrophil Cells | 0.783216783 | 0.002586215 |
| Eosinophil Cells | -0.712875173 | 0.00926089 |
| B Cells Memory | -0.112084235 | 0.728736104 |
| B Cells Naive | -0.168126352 | 0.601453777 |
| Plasma Cells | -0.307692308 | 0.330589259 |
| T Cells CD8 Actived | -0.005375567 | 0.986771575 |
| T Cells CD8 Naive | 0.257010559 | 0.420009062 |
| T Cells CD8 Memory | -0.421091835 | 0.172809961 |
| M0 Macrophage | -0.723919953 | 0.007769831 |
| M1 Macrophage | 0.41958042 | 0.174519008 |
| M2 Macrophage | 0.238471918 | 0.45541804 |
| Treg Cells | -0.454545455 | 0.137657916 |
| T Cells CD4 Memory | -0.524475524 | 0.080019376 |
| T Cells CD4 Naive | 0.238178999 | 0.455988553 |
| T Cells CD4 Follicular | NA | NA |
| Th1 Cells | -0.080560544 | 0.803451109 |
| Th17 Cells | -0.356643357 | 0.255137752 |
| Th2 Cells | NA | NA |
| Monocyte | 0.188811189 | 0.556737056 |
| GammaDelta T Cells | -0.657342657 | 0.020185499 |
| NK Resting | 0.05225263 | 0.87187652 |

| **Table S8 Results of the correlation analysis between Slc39a14 and immune cells.** | | |
| --- | --- | --- |
| cell | cor | p.value |
| Mast Cells | 0.041958042 | 0.896985871 |
| Neutrophil Cells | 0.923076923 | 1.86E-05 |
| Eosinophil Cells | -0.69794585 | 0.011603191 |
| B Cells Memory | -0.150613191 | 0.640334525 |
| B Cells Naive | -0.185639514 | 0.563504136 |
| Plasma Cells | -0.300699301 | 0.342259529 |
| T Cells CD8 Actived | 0.112886912 | 0.726858861 |
| T Cells CD8 Naive | 0.201936868 | 0.529093491 |
| T Cells CD8 Memory | -0.483475811 | 0.111294307 |
| M0 Macrophage | -0.58246433 | 0.046889974 |
| M1 Macrophage | 0.370629371 | 0.235621004 |
| M2 Macrophage | 0.156608424 | 0.626924802 |
| Treg Cells | -0.545454545 | 0.066611882 |
| T Cells CD4 Memory | -0.097902098 | 0.762121656 |
| T Cells CD4 Naive | 0.143607926 | 0.656129207 |
| T Cells CD4 Follicular | NA | NA |
| Th1 Cells | -0.143607926 | 0.656129207 |
| Th17 Cells | -0.433566434 | 0.159105769 |
| Th2 Cells | NA | NA |
| Monocyte | 0.132867133 | 0.680597508 |
| GammaDelta T Cells | -0.587412587 | 0.044609296 |
| NK Resting | 0.0485203 | 0.88096859 |

**Table S9 Pearson analysis with SLC7A11 levels in BALF**

|  | **r** | **95% confidence interval** | **R^2^** | ***p* value** |
| --- | --- | --- | --- | --- |
| **Neutrophils, %** | 0.2935 | 0.02526 to 0.5224 | 0.08617 | 0.0329 |
| **ICU duration, days** | -0.2408 | -0.4778 to 0.02879 | 0.05800 | 0.0794 |
| **Leukocytes, %** | -0.2364 | -0.4762 to 0.03625 | 0.05587 | 0.0884 |
| **Lymphocyte, %** | -0.06486 | -0.3294 to 0.2091 | 0.004206 | 0.6445 |
| **PCT, ng/mL** | 0.3931 | 0.1374 to 0.5997 | 0.1545 | 0.0036 |
| **CD3%** | -0.3397 | -0.6596 to 0.08427 | 0.1154 | 0.1127 |
| **CD8%** | -0.3458 | -0.6634 to 0.07745 | 0.1196 | 0.1061 |
| **CD4%** | -0.1353 | -0.5186 to 0.2933 | 0.01830 | 0.5383 |
| **CD4/CD8** | 0.2280 | -0.2033 to 0.5852 | 0.05199 | 0.2954 |
| **CD3, /uL** | -0.4253 | -0.7126 to -0.01594 | 0.1809 | 0.0430 |
| **CD4, /uL** | -0.3490 | -0.6655 to 0.07382 | 0.1218 | 0.1026 |
| **CD8, /uL** | -0.4889 | -0.7499 to -0.09602 | 0.2390 | 0.0179 |
| **Monocyte, %** | -0.1176 | -0.5137 to 0.3199 | 0.01383 | 0.6022 |
| **Eosinophils, %** | 0.04420 | -0.3950 to 0.4670 | 0.001954 | 0.8491 |
| **Monocytes, 10^9^/L** | -0.3792 | -0.6904 to 0.05048 | 0.1438 | 0.0818 |
| **Eosinophils, 10^9^/L** | -0.2910 | -0.6420 to 0.1609 | 0.08467 | 0.2006 |
| **CRP, ng/L** | 0.1601 | -0.2805 to 0.5449 | 0.02562 | 0.4767 |
| **IL-6, ng/L** | 0.02558 | -0.4003 to 0.4424 | 0.0006543 | 0.9100 |
| **Neutrophils, 10^9^/L** | -0.1378 | -0.5287 to 0.3013 | 0.01898 | 0.5409 |
| **Lymphocyte, 10^9^/L** | -0.4174 | -0.7134 to 0.005167 | 0.1742 | 0.0533 |
